# Using surface-enhanced Raman scattering for simultaneous multiplex detection and quantification of thiols associated to axillary malodour†

**DOI:** 10.1039/d4an00762j

**Published:** 2024-06-24

**Authors:** Amy Colleran, Cassio Lima, Yun Xu, Allen Millichope, Stephanie Murray, Royston Goodacre

**Affiliations:** a Centre for Metabolomics Research, Department of Biochemistry, Cell and Systems Biology, Institute of Systems, Molecular and Integrative Biology, University of Liverpool Crown St Liverpool L69 7ZB UK roy.goodacre@liverpool.ac.uk; b Unilever Research and Development Port Sunlight Bebington CH63 3JW UK

## Abstract

Axillary malodour is caused by the microbial conversion of human-derived precursors to volatile organic compounds. Thiols strongly contribute to this odour but are hard to detect as they are present at low concentrations. Additionally, thiols are highly volatile and small making sampling and quantification difficult, including by gas chromatography-mass spectrometry. In this study, surface-enhanced Raman scattering (SERS), combined with chemometrics, was utilised to simultaneously quantify four malodourous thiols associated with axillary odour, both in individual and multiplex solutions. Univariate and multivariate methods of partial least squares regression (PLS-R) were used to calculate the limit of detection (LoD) and results compared. Both methods yielded comparable LoD values, with LoDs using PLS-R ranging from 0.0227 ppm to 0.0153 ppm for the thiols studied. These thiols were then examined and quantified simultaneously in 120 mixtures using PLS-R. The resultant models showed high linearity (*Q*^2^ values between 0.9712 and 0.9827 for both PLS-1 and PLS-2) and low values of root mean squared error of predictions (0.0359 ppm and 0.0459 ppm for PLS-1 and PLS-2, respectively). To test this approach further, these models were challenged with 15 new blind test samples, collected independently from the initial samples. This test demonstrated that SERS combined with PLS-R could be used to predict the unknown concentrations of these thiols in a mixture. These results display the ability of SERS for the simultaneous multiplex detection and quantification of analytes and its potential for future development for detecting gaseous thiols produced from skin and other body sites.

## Abstract 2

SERS in combination with chemometric techniques has been used to simultaneously detect and quantify malodourous thiols which are associated with axillary malodour in multiplex solutions.

## Graphical abstract

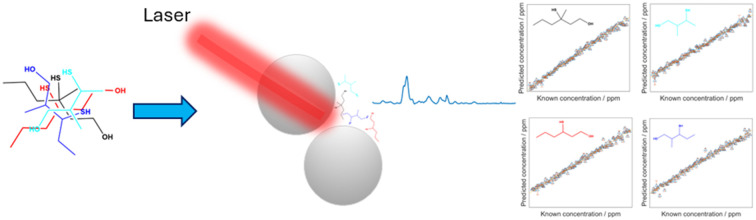

## Introduction

Human body odour is a form of social communication that can provide a wide array of information to another person. This includes health, emotional status, sex, age or ethnicity of a person.^1,2^ However, unpleasant body odour can be perceived in a negative light due to being associated with personal hygiene. These unpleasant smells can impact interactions between people, as well as the social standing and confidence of the individual affected.^3,4^ One of the main unpleasant odours produced by the body is axillary malodour.

Since the 1950s, the biotransformation of odourless precursor molecules from apocrine glands, located on the axilla skin, to malodorous volatile organic compounds (VOCs) by microbial communities surrounding these glands, has been widely accepted as the cause of axillary malodour.^5,6^ Staphylococci, in particular *Staphylococcus hominis*, are of particular interest in contributing to the production of malodorous compounds.^6–10^ However, other genera are also found to be present and these may also contribute to malodour production.^8,11–14^ The predominant malodorous VOCs produced in the axilla are thiols, with the generation of volatile fatty acids (VFAs) as a further contributary factor in axillary malodour.^14^

Thiols can be highly pungent and contribute strongly to the odour of the axilla. The four thiols, which are analysed in this study and have been detected as axillary malodourants are 3-mercaptohexan-1-ol (3MH), 2-methyl-3-mercaptobutan-1-ol (2M3MB), 2-methyl-3-mercaptopentan-1-ol (2M3MP), and 3-methyl-3-mercaptohexan-1-ol (3M3MH), with the latter being the most abundant thiol naturally present out of the group being investigated. However, despite their low abundance, the human olfactory system has an extremely low threshold of detection for these thiols, being at pg L^−1^ in the air.^6,15,16^ In *S. hominis* and other *Staphylococcus* species, 3M3MH has been shown to be produced through the breakdown of Cys-Gly-3M3MH to 3M3MH through the action of a bacterial dipeptidase. This is followed by a staphylococcal specific C–S β-lyase enzyme to break the C–S bond and release 3M3MH.^9^ All other volatile malodorous thiols are believed to follow the same pathway, from a cysteinylglycine conjugate of the thiol to the malodorous free thiol.^7,17,18^

These free thiols are naturally present at low concentration levels. Additionally, they have a low molecular weight and are highly volatile. This makes them very difficult to quantify and analyse. Subsequently, little information has been gained about these compounds.^19^ As key biomarkers of bacterial metabolism, and the primary causative agent in the generation of axillary malodour, the accurate detection and quantification of these thiol-based odorants is essential for efficacy testing of cosmetic products such as deodorants and anti-perspirants, designed to reduce thiol generation.^20–22^

Currently, the main methods involved for detecting and quantifying these thiols and VFAs is by GC-MS.^12,15,23–25^ In previous research, GC-MS has sometimes been used with a sniff port attached and using volunteers for qualitative detection.^6,26,27^ GC-MS is used due to its high sensitivity, selectivity and being able to examine the data quantitatively. There are established methods for VOC detection, resulting in good reproducibility. Additionally, with large reference libraries available for GC-MS, it is considered a highly accurate technique. However, this method is expensive, time-consuming, and sample preparation can be complex.^28,29^ Moreover, measurements of the odorous thiols are generated following solvent extraction and reacidification of solid phase microextraction of the axilla sweat samples.^6,15,30–32^ Consequently, the detection is not real time and molecules of interest may be lost during sample preparation.^33^ Hence, it would be advantageous to track the production of malodorous thiols and correlate them with matched analysis of the abundance of bacterial species present using analytical methods that are accurate, affordable, sensitive to volatile small molecules, and usable for real-time and point-of-care analysis. Raman scattering methods, in particular surface-enhanced Raman scattering (SERS), is an area of analytical techniques that has this potential.

SERS is a non-destructive vibrational spectroscopic method that enhances the weak Raman signal produced from the interactions of light with the molecular vibrations of an analyte, for identification and quantification. The signal is enhanced by using roughened metallic substrates (usually gold or silver) such as colloidal nanoparticles. The advantage of using this technique for detecting thiols is the chemisorption of sulfur present in the thiols to the metal surface and the formation of self-assembled monolayers (SAMs), leading to greater enhancement of the Raman signal.^34–36^ Moreover, this method can be used for real time and point-of-care analysis due to the availability of portable Raman devices. As a result, SERS has been used to detect and quantify a wide range of thiols and sulfur compounds, including volatile sulfur compounds.^37–43^ Additionally, sulfur compounds can be used as reporter and linking molecules for SERS to detect an analyte of interest.^44–47^

In this study, colloidal nanoparticles in suspension were used to detect and quantify four thiols associated to axillary malodour, both individually and simultaneously, in a four-plex mixture using SERS. The limit of detection values of the thiols was evaluated and compared using both univariate and multivariate techniques. Based on these results, multivariate analysis was used to quantify the thiols in a mixture of different concentrations of thiols. The developed multivariate model was applied to a blind test set to estimate unknown thiol concentrations in mixtures. Out of the four thiols, only 3MH had been previously analysed using SERS, therefore this study shows the SERS spectra of these other thiols for the first time.^48^ Moreover, this research is one of a limited number of studies that has performed quantitative multiplex analysis and predict the unknown concentrations of analytes simultaneously in a sample using SERS. Thiols were analysed as liquids rather than gases in this study. This is because, as previously stated, three of these thiols had not previously been detected using SERS, and additionally, it is easier to initially examine the analytes as a liquid rather than a gas.

## Methods

### Materials

Trisodium citrate, gold(iii) chloride hydrate (99.999%), sodium hydroxide, hydroxylamine hydrochloride, acetonitrile and sodium chloride were purchased from Fisher Scientific (Loughborough, UK). Hydrochloric acid, nitric acid and silver nitrate were purchased from Sigma Aldrich Ltd (Dorset, UK). 3-Mercapto-hexan-1-ol (96%) was purchased from Alfa Aesar (Heysham, UK) and 3-methyl-3-mercapto-hexan-1-ol, 2-methyl-3-mercapto-butan-1-ol and 2-methyl-3-mercapto-pentan-1-ol were all purchased from and synthesised by Key Organics Ltd (Cornwall, UK).

### Gold and silver nanoparticle synthesis

All glassware used for the synthesis of silver nanoparticles was cleaned with aqua regia (HNO_3_ : HCl, 1 : 3, v/v) to remove any residual metals, followed by thoroughly rinsing with deionised water. The glassware was dried and sterilised through autoclaving before use.

### Synthesis of hydroxylamine-stabilised silver nanoparticles (hAgNPs)

Hydroxylamine-stabilised silver nanoparticles were synthesised by adopting the Leopold and Lendl method.^49^ 20 mL of sodium hydroxide solution (30 mM) was dissolved into 158 mL of deionised water, under continuous stirring, followed by 2 mL hydroxylamine hydrochloride solution (150 mM). To this solution, 20 mL of diluted silver nitrate solution (10 mM) was slowly added dropwise to the sodium hydroxide/water solution. The solution was left stirring for 15 min. A change in colour of the solution was observed from colourless to an orange/grey which indicated successful nanoparticle formation. This was repeated four more times to produce five batches of hydroxylamine-stabilised silver nanoparticles to examine the reproducibility of the SERS signal by the nanoparticles.

### Synthesis of citrate-stabilised gold nanoparticles (cAuNPs)

Citrate-stabilised gold nanoparticles were synthesised using the Lee and Meisel method.^50^ Chloroauric acid (0.24 g) was dissolved into 500 mL of deionised water and brought to the boil, with continuous stirring. Trisodium citrate (1.0 g) was dissolved in 100 mL deionised water and 50 mL of trisodium citrate solution was added dropwise, slowly to the chloroauric acid/water solution. The solution was left to boil for 1 h and left to return to room temperature. A change in colour of the solution was observed from yellow to a dark red/purple indicating successful nanoparticle formation.

### Synthesis of citrate-stabilised silver nanoparticles (cAgNPs)

Citrate-stabilised silver nanoparticles were synthesised using the Lee and Meisel method.^50^ Silver nitrate (0.09 g) was dissolved into 500 mL of deionised water and brought to the boil, under continuous stirring. Trisodium citrate (1.0 g) was dissolved in 100 mL deionised water and 10 mL of trisodium citrate solution was added dropwise, slowly to the silver nitrate/water solution. The solution was left to boil for 1 h and left to return to room temperature. A change in colour of the solution was observed from colourless to a milky green/grey suspension indicating successful nanoparticle formation.

All nanoparticles synthesised were characterised using UV-Vis, zeta-potential and scanning electron microscopy. The results of the characterisation and reproducibility studies of hAgNPs can be found in ESI 1.†

### Sample preparation for SERS measurements of individual thiols and thiols in a multiplex solution

For measuring the limit of detection of each of the thiols, individual samples were prepared by dissolving stock solutions of each thiol (10 000 ppm in 50% acetonitrile) to concentrations between 0.0058 ppm and 3 ppm in an aqueous solution, adjusted to pH 11 using 1 M NaOH.

Following the determination of the limit of detection (LoDs) and the total concentration at which one monolayer of analyte on the nanoparticle surface is exceeded, 120 multiplex samples of all four thiols were produced using latin hypercube sampling (LHS) to determine the concentration of each thiol in the sample (ESI 2†).^51^ The concentrations used were between 0.05 ppm and 1 ppm for each thiol in each sample.

**Fig. 1:**
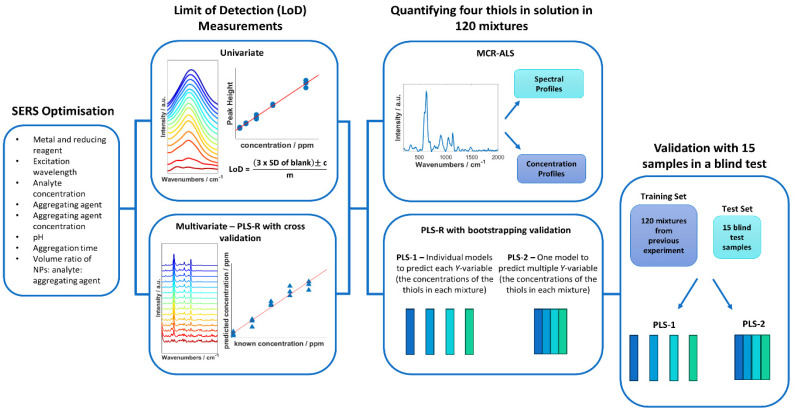
Overview of the steps taken in this study to detect and quantify malodorous thiols.

Blind test sampling of the multiplex solutions was performed with 15 new samples consisting of randomly generated concentrations for each thiol using the same concentration >ranges as the previous multiplex solutions. The concentrations used were generated with a random number generator in Matlab R2021a. The 15 thiol samples were formulated in Matlab and prepared by one analyst. A second analyst prepared the SERS samples, then measured and performed PLS-R on the samples, without any prior knowledge to the concentrations in the mixtures (Fig. 1).

### SERS measurements

SERS analyses were performed with a DeltaNu Advantage 200A Raman spectrometer (DeltaNu, Laramie, WY, USA) using a 785 nm laser with a typical power on the sample of ∼60 mW. Spectra were acquired over 200 to 2000 cm^−1^. Prior to any measurements, SERS parameters were optimised. The optimised conditions used are listed in Table 1. All SERS samples were prepared using the following optimised parameters: 270 μL of hAgNPs were transferred to a glass vial, followed by 130 μL of thiol solution at the correct concentration and 50 μL of NaCl (0.2 M). The mixture was vortexed for 10 s and a spectrum was acquired immediately for 30 s. Three replicates were prepared for each sample in all experiments.

**Table 1:** Optimised SERS parameters used for measuring thiols with hydroxylamine-stabilised silver nanoparticles (hAgNPs)

Excitation wavelength	785 nm
Acquisition time	30 s
Nanoparticles	hAgNPs
Aggregating agent	NaCl
Aggregating agent concentration	0.2 M
Volume ratio (hAgNPs : analyte : aggregating agent)	270 μL : 130 μL : 50 μL
pH	11
Aggregation time	30 s

### Data analysis

All SERS data were processed using Matlab R2021a (The MathWorks, Natick, MA, USA). For principal component analysis (PCA), spectra were first baseline corrected using asymmetric least squares, smoothed with a Savitzky–Golay filter with a 2nd order polynomial and a window width of 11 points and vector normalised.^52–54^ For univariate calculations of limit of detection (LoD), spectra were left untreated, however, individual peaks were chosen for the calculations and baseline corrected before measuring peak heights. For all PLS-R analysis, spectra were vector-normalised only, with no further pre-processing which could lead to overfitting of the data. For multivariate curve resolution-alternating least squares (MCR-ALS), spectra were normalised using standard normal variate (SNV) normalisation only. The first derivative of the normalised spectra was calculated and used for MCR-ALS.

For calculating the limit of detection of the individual thiols, both univariate and multivariate methods were used. Initially, PCA was carried out as an unsupervised method to reduce the dimensionality of the SERS spectra.^55^ Score plots were used to visualise the changes in the spectra with decreasing concentration. Loadings plots of PC-1 were used to determine suitable peaks from to use for univariate calculations of the limit of detection as well as the linearity of the results of the five lowest concentrations for the chosen peaks used to calculate the LoD. The peak heights of the chosen peaks were used for univariate analysis. Calibration curves were constructed using the peak heights and the following equation was applied to calculate the limit of detection from the linear region of the calibration plot:1
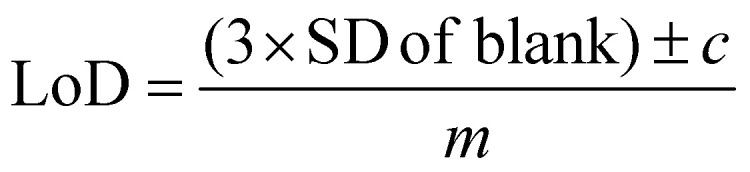
where SD is the standard deviation, *c* is the *y*-intercept and *m* is the slope.^56^

Partial least squares regression (PLS-R) was used as a multivariate analysis method to analyse the relationship between the SERS spectra of the analytes and the concentrations used and to determine the LoD.^57^ The models were validated by leave one concentration out double cross validation. The limit of detection was calculated using the experimental concentrations and the concentrations predicted by PLS-R with the method.^58^

To build a concentration prediction model from the 120 multiplex samples, PLS-R was used with 1000 bootstrapping resampling as the validation method, and all plots show the predictions on the 1000 test sets only.^59^ Two PLS-R modelling methods were used to predict the concentrations: PLS-1 which creates prediction models from each thiol's individual concentrations and PLS-2 which creates prediction models simultaneously from all thiol concentrations in the samples in one matrix.^60^ The trained PLS-1 and PLS-2 models were used for predicting the concentrations of thiols from the SERS spectra obtained from the 15 blind test samples.

In addition to PLS-R models, multivariate curve resolution with alternative least square (MCR-ALS) was also applied to multiplex samples. MCR-ALS attempts to decompose mixed spectra of multiplex samples into concentration profiles and spectral profiles. The spectral profiles represent the “recovered” pure spectra of the thiols in the mixture while the concentration profiles represent the contribution of each of the spectrum which in turn correlates the concentrations of these thiols in the mixture. Subsequently, four linear regression models, one for each thiol, were built between the concentration profile of each thiol and the known concentration of the corresponding thiol.

## Results and discussion

### SERS spectra of thiols individually and in a mixture

Thiols are a highly suitable analyte for analysing with SERS due to the formation of a metal-sulfur bond, leading to chemical enhancement of the thiol.^61^ 3-Mercapto-hexan-1-ol (3MH), 3-methyl-3-mercapto-hexan-1-ol (3M3MH), 2-methyl-3-mercapto-butan-1-ol (2M3MB) and 2-methyl-3-mercapto-pentan-1-ol (2M3MP) are odorous thiols associated to axilla odour. Some of these thiols can also be associated to food and drink odours such as the presence of 3MH as a fruity odour in wine and passionfruit, the presence of 2M3MB and 2M3MP in onions and 2M3MB in meaty flavouring in processed foods.^62–66^ However, out of the four thiols tested, only 3MH has previously been analysed as a flavour in wine using SERS with functionalised silver nanocubes on Si wafers.^48^ None of these thiols have been analysed with nanoparticles in solution, either individually or in a mixture with one another. It was therefore important initially to optimise SERS parameters to produce the greatest and most reproducible enhancement of the SERS spectra of the thiols. The parameters for three distinct types of nanoparticles were optimised for 3MH and these conditions were then tested on the other three thiols. The final optimised conditions were applied to all further experiments and are found in Table 1 (further details on SERS optimisation experiments can be found in the ESI†).

**Fig. 2:**
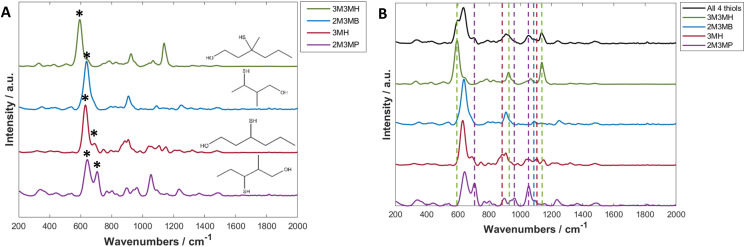
(A) Average SERS spectra of 3M2MP, 3M2MB, 3M3MH and 3MH at 3 ppm (B). Average SERS spectra of 3M2MP, 3M2MB, 3M3MH and 3MH at 3 ppm and all thiols in a mixture, each at 1 ppm in 50% acetonitrile solution using hydroxylamine-stabilised silver nanoparticles at pH 11 with NaCl (0.2 M) used as an aggregating agent. The spectra are the averages of 3 measurements and have been baseline corrected, smoothed and vector normalised. Asterisks in A highlight the peaks that are associated to the *trans* and *gauche v*(C–S) bands. Coloured dashed lines in (B) highlight the bands that are found in the spectrum of multiple thiols that are found in the spectra of the individual thiols. The colours of the lines are the same as the colours used for the individual thiol spectrums to indicate which band in the mixture spectrum is associated to which thiol.

The spectra of the individual thiols and thiols at the same concentration in a mixture using these parameters are shown in Fig. 2. For all the spectra of the individual thiols, there is one peak between 590 to 645 cm^−1^ which is the most intense (Fig. 2A). When comparing this peak to other SERS spectra of different thiols and of the SERS spectra of 3MH previously analysed, this is the carbon – sulfur stretching vibrational mode (*v*(C–S)) band.^48,61^ The high intensity of the peak is due to the bond being closest to the nanoparticle surface due to the binding of the sulfur to the silver nanoparticles. Therefore, there is likely to be some enhancement from chemical interactions present between the thiols and the nanoparticles, as well as electromagnetic enhancement mechanisms. The second peak seen in the spectra for 2M3MP and 3MH at 706 and 673 cm^−1^ respectively is likely to be another *v*(C–S) band for the *trans* conformation of the thiol. Whereas, the more intense peak for all the thiols is likely to be the *gauche* conformation.^36,61,67^ This could be because, compared to other thiols that have been analysed previously, the sulfur is located in the centre of the carbon chain instead of at the end. Therefore, there is likely a greater amount of steric hinderance between the carbon chains and methyl groups in the *trans* position compared to the *gauche* position, in relation to the position of the sulfur group.

As this study was interested in eventually being able to detect all four thiols in a mixture, it was important to examine the spectra of all four thiols at the same concentration to see if peaks in the spectrum of the mixture could be visually associated to individual thiols. For all thiols, certain peaks in the spectrum of the mixture can clearly be identified as relating to that thiol (Fig. 2B and Table 2). Examples of this include the *v*(C–S) band at 596 cm^−1^ for 3M3MH and the *trans v*(C–S) band at 706 cm^−1^ for 2M3MP. Therefore, even without any initial chemometrics applied to the data, there is some confirmation of different thiols being present in the mixture and the intensity of these peaks did vary with changes in concentration in the mixtures (see ESI and Fig. S15†). This initial analysis of thiols at the same concentration in a mixture indicated that there would be a good chance of detection and quantification of these thiols in a mixture with varying concentrations.

**Table 2:** Table of characteristic bands found in the spectrum of all four thiols in a mixture and the thiol each peak is associated to

Band in spectrum of all four thiols in a mixture (cm^−1^)	Thiol assignment
596	3M3MH
706	2M3MP
887	3MH
926	3M3MH
963	2M3MP
1053	2M3MP
1089	2M3MB
1107	3MH
1137	3M3MH

### Limit of detection (LoD) measurements of the individual thiols using univariate and multivariate analysis

After the optimised SERS parameters were found, limit of detection (LoD) studies were carried out on each of the thiols individually. This was completed by diluting the 10 000 ppm stock solutions in aqueous solutions adjusted to pH 11 with 1 M NaOH to concentrations between 3 ppm and 0.0058 ppm. Two methods were used for calculating the limit of detection. The first is a univariate method which calculates what the lowest concentration is in which the peak height of a peak related to the analyte is greater than 3 times the standard deviation of the signals at the same region in the blank sample.^56,68^ The second is a multivariate method, PLS-R, which calculates the limit of detection from looking at the relationship between the known concentration of the solution and the concentration predicted by the PLS-R model.^69^

### Univariate analysis

Univariate analysis involves looking at the changes of the properties of one variable (such as a peak at a certain wavenumber) to produce a calibration model with a dependent variable (such as the concentration) which could then be used to predict the dependent variable of an unknown sample.^70^

**Fig. 3:**
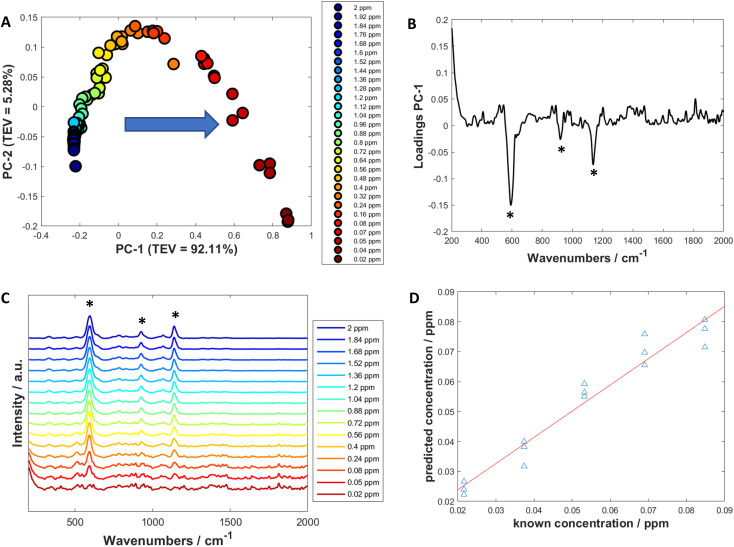
(A) A PCA scores plot of 3M3MH at varying concentrations between 0.02 ppm and 2 ppm. The arrow indicates direction of decreasing concentration and TEV is the total explained variance. (B) Loadings plot of PC-1. (C) Spectra of 3M3MH varying in concentration between 0.02 ppm (red) and 2 ppm (blue). (D) PLS-R prediction plots of the test data for 3M3MH and used to calculate LoD using multivariate calculations. Asterisks in (B and C) represent the peaks chosen for the univariate calculation of the limit of detection (LoD). PLS-R models were produced using leave-one-out double cross validation. Data for figures (A–C) has been baseline corrected, smoothed and vector normalised.

Initially, to examine how the spectra changed with decreasing concentration, PCA was used to visualise and determine which peaks showed the biggest change and therefore which peaks to use for calculating limit of detection (Fig. 3A and Fig. S11A, S12A and S13A†). The PCA score plots demonstrated a trend in PC-1 axis from negative to positive score values as the concentration decreased. This was the dominant trend in all the scores plots, with PC-1 showing high values of total explained variance (TEV), such as 92.11% TEV for PC-1 for 3M3MH score plot (Fig. 3A). Examination of the loadings plots for PC-1 displayed the peaks which were greatest in intensity for the thiol of interest and showed the most negative score values (Fig. 3B). For 3M3MH, there were three peaks at 596 cm^−1^, 920 cm^−1^ and 1139 cm^−1^ (highlighted with asterisks). These peaks were then used to determine the LoD for each of the thiols. The peak which contributed to the most variance in PC-1 for all the thiols was the *v*(C–S) band (Fig. 3B and Fig. S11B, S12B and S13B†). This is to be expected as the sulfur chemisorbs to the metal surface and produces the strongest intensity out of all the vibrational modes for the thiols. The lowest five concentrations, in the linear regions of these calibration curves, were used to calculate the LoD using eqn (1). These were used as the linear region should have a minimum of five datapoints within the linear region of the LoD for the LoD to be calculated. This calculation therefore reflects the uncertainty of this measurement in the low concentration range.^68^

For all four thiols tested, there were differences in both linearity and in LoD values between the peaks chosen for each thiol. This was due to differences in the linear regions of the calibration curves. For three of the thiols, 3MH, 2M3MB and 2M3MP, the *gauche v*(C–S) bands of each of the thiols were found to have the lowest limits of detection, with values between 2.77 × 10^−2^ ppm for 3M3MH and 1.14 × 10^−2^ ppm for 3MH (Table 3).

**Table 3:** LoD measurements of 3M3MH, 3MH, 2M3MB and 2M3MP calculated using peak heights highlighted in PC-1 loadings plots. *R*^2^ represents the goodness of fit of the peak heights in the calibration model to known concentrations

Wavenumber (cm^−1^)	*R* ^2^	LoD (ppm)
**3M3MH**		
591	0.8804	0.0277
920	0.7253	0.0872
1139	0.8279	0.0170
**2M3MB**		
639	0.9889	0.0212
910	0.9221	0.5004
**3MH**		
634	0.9070	0.0114
**2M3MP**		
642	0.8853	0.0137
706	0.8631	0.024
1234	0.7567	0.1741

The reason the *v*(C–S) band can be detected at a very low concentration is because the signal-to-noise ratio of this peak is higher than the other peaks in the spectra. As previously discussed, the *v*(C–S) band is the most intense as the C–S bond is very close to the metallic surface. It is therefore strongly enhanced through chemical and electromagnetic enhancement. The thiol which, overall, showed the best linearity appeared to be 2M3MB whereas the thiol with the lowest overall LoD values appeared to be 3M3MH.

### Multivariate analysis

A SERS spectrum of an analyte contains many peaks which will all vary with changing concentrations. This implies there are multiple variables to contend with. This makes SERS spectra suitable for quantification using multivariate methods. For these experiments, PLS-R was employed to produce these quantitative models and to determine LoD. PLS-R involves predicting the concentration of the thiol of interest from the corresponding spectra.^71^

PLS-R plots were created using the same five solutions used for univariate analysis (Fig. 3D and Fig. S11D, S12D and S13D†) with leave-one-out double cross validation to assess the reproducibility and validity of the models. *Q*^2^ (a correlation coefficient of the predicted and known concentrations of the test data) showed values for all thiols of between 0.74 and 0.985 (Table 4). 3MH shows a much lower value of *Q*^2^ compared to the other thiols. This is due to an outlying datapoint being predicted as a much lower concentration by the model (Fig. S12D†). Nevertheless, there is good agreement between the predicted and known concentrations for each of the thiols. Additionally, the root mean square error on the cross validation (RMSECV) was performed to calculate the amount of error in the predicted concentrations. For the thiols, RMSECV values were between 0.0053 and 0.0083 ppm. 2M3MB showed the best fit and 3M3MH showed the lowest RMSECV value when comparing all the thiols. The LoDs were calculated using PLS-R with the method described by Ortiz *et al.*^69^ The values were found to be between 0.0227 ppm (2M3MP) and 0.0153 ppm (2M3MB). These values for LoD lie within the values found for the peak heights using univariate analysis.

**Table 4:** LoD calculated for each of the thiols using PLS-R modelling. Where *Q*^2^ is the goodness of fit of the predicted concentrations from the test data to the known concentrations and RMSECV is the root mean square error of cross validation

Thiol	LoD (ppm)	*Q* ^2^	RMSECV (ppm)
3M3MH	0.0174	0.9431	0.0053
2M3MB	0.0153	0.9850	0.0070
3MH	0.0209	0.7482	0.0083
2M3MP	0.0227	0.8867	0.0074

As previously discussed, when using univariate analysis, the LoDs are calculated for the individual peaks of each thiol. This explains why multiple LODs are reported in Table 3. Therefore, if one peak is more intense at higher concentrations compared to the other peaks present within the spectrum, it is likely that this peak will show a greater signal-to-noise ratio (SNR) at much lower concentrations compared to peaks which start at lower intensities. Consequently, this can lead to widespread LoD estimates for each analyte. Additionally, to be able to perform quantitative predictive analysis with univariate methods, there can be no interference from any other variable present when measuring the variable of interest. Whereas, when using multivariate methods, the whole spectrum is used to calculate the LoD, so there is no need for selectivity of peaks within the data. Therefore, for SERS spectra containing multiple peaks of interest, when measuring the LoD, multivariate techniques can be less time consuming and can improve the visualisation and quantification of the relationship between predictive and known variables.

Overall, in comparison to gas chromatography, SERS is less sensitive, with 3M3MH being detectable in incubated sweat using gas chromatography with an atomic emission detector at around 4 ppb and could have potentially been lower, although, this is not stated.^19^ Furthermore, using direct immersion SPME GC-MS, the LoD of 3M3MH was recorded as 0.06 ng mL^−1^ (0.06 ppb).^72^ It is likely the detectability of the other three thiols using both methods is similar to 3M3MH. However, as previously discussed, these methods require further extraction and concentration to produce such low LoDs. SERS did not require any further processing to the samples, making the analysis quicker and easier to perform.

### Multiplex detection and quantification of thiols

After the LoDs were established for all four thiols, the next step was to investigate if the thiols could be detected and quantified in a mixture of all four thiols at varying concentrations. Before trying this however, mixtures of two thiols in solution at equal concentration at various total concentrations were measured. This was performed to find the total concentration at which there was no longer a monolayer of thiols on the surface of nanoparticles. This was important as at total concentrations higher than one monolayer, the more thermodynamically favoured thiol will cover the surface of the nanoparticles due to competition for the metal surface. Equal amounts of different thiols will only be seen on the surface of nanoparticles at concentrations of one monolayer or less.^44,73^ The results from these measurements and further information can be found in ESI 1.† However, from examining the plots showing changes in peak area, it was found that if the total concentrations were kept below 20 ppm, both thiols could be detected in solution and above 20 ppm, changes were seen in the peak areas.

**Fig. 4:**
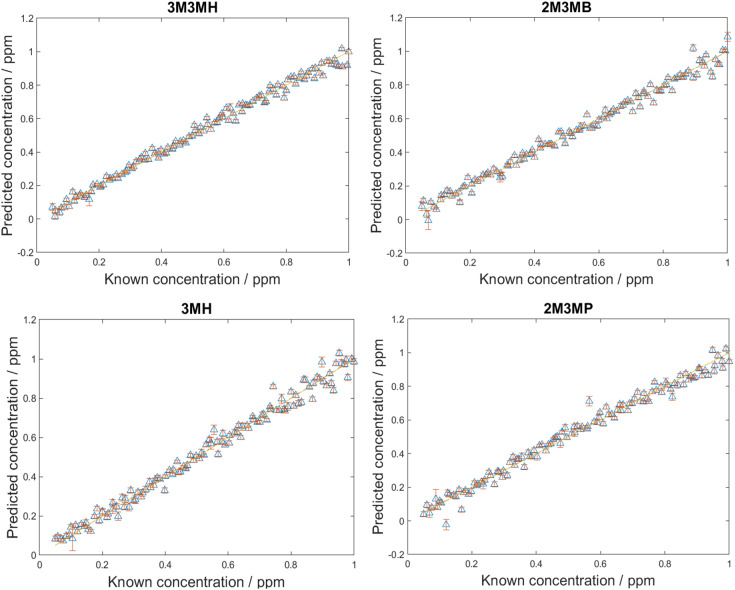
PLS-2 prediction plots of the test data for each of the thiols of interest in multiplex mixtures. PLS-R models were produced using 1000 bootstraps for validation. Points are averages with standard deviation error bars for these 1000 data resamplings.

For the detection and quantification of thiols in a mixture, 120 mixtures were produced using Latin Hypercube Sampling (LHS) experimental design (details provided in ESI 2†). The concentration of each thiol in each mixture was between 0.05 ppm and 1 ppm. This range ensured that the concentrations were above the LoD, so all thiols should be detectable within this range. PLS-R modelling was then applied to the spectra with 1000 bootstraps for re-sampling. At each bootstrap repetition, a few samples were chosen as the training set and the rest as the validation set. The samples were replaced, and this process was repeated over the number of iterations (in this case, 1000) to examine the performance of the model to predict the parameter of interest. In this case, it was to predict the concentrations of the thiols in the mixtures. Two different PLS-R models were used to interpret the data, PLS-1 and PLS-2. PLS-1 creates four individual models for each thiol concentration set separately (*i.e.*, four models with a single *Y*-variable being predicted) while PLS-2 creates models for each thiol concentration set by looking at all four thiol concentrations simultaneously (*i.e.*, a single models predicting four *Y*-variable at the same time). As PLS-2 attempts to model the outputs for all four thiols simultaneously, as previously observed, this can lead to greater variation in the predictions of the concentrations, poorer values for *Q*^2^ and greater values of root mean square error (RMSE).^60,74^ However, in the case of these thiol mixtures, the results seen for both PLS-1 and PLS-2 were remarkably similar (Fig. 4, Table 5). There is excellent linearity displayed for both the training and test sets, with *R*^2^ values for both PLS-1 and PLS-2 models for all thiols greater than 0.97 and *Q*^2^ (CV) and *Q*^2^ (test) greater than 0.96. Furthermore, the RMSEC values were all less than 0.046 ppm, RMSECV values were all less than 0.052 ppm and RMSEP values were all less than 0.046 ppm for both PLS-1 and PLS-2 models. The good agreement between the RMSEC, RMSECV and RMSEP values suggests the models are stable. Moreover, this shows that for the training, validation and test sets, there were low prediction errors and excellent linearities for predicting concentrations of the thiols in the multiplex mixtures. This was seen particularly with the RMSEP values, which showed minimal difference between PLS-1 and PLS-2 and for three of the thiols, PLS-2 showed lower RMSEP values than PLS-1. However, to achieve these results, the PLS-2 model used more latent variables (11 latent variables) than the individual PLS-1 models (6, 7, 8 and 6 for 3M3MH, 2M3MB, 3MH and 2M3MP respectively). This is due to the increased complexity of having a matrix of four *Y*-variables and therefore more latent variables are needed for PLS-2 for low values of RMSEP. When comparing the results for the thiols across both models, 3M3MH appeared to show better results for linearity and low RMSEP. These results were comprehendible from Fig. 2. In the spectrum of the mixture, there were a number of peaks that can only be associated to the spectrum for 3M3MH, particularly the peak at 596 cm^−1^. On the other hand, the SERS spectra of 3MH, 2M3MP and 2M3MB show more peaks at similar wavenumbers to one another and with 3M3MH making them harder to distinguish by eye in the spectrum of the mixture.

**Table 5:** PLS-1 and PLS-2 model results for measuring all four thiols in a mixture at different concentrations. Where *R*^2^, *Q*^2^ (CV) and *Q*^2^ (test) are the correlation coefficients of the predicted and known concentrations for the training, cross validation, and test sets, respectively. RMSEC, RMSECV and RMSEP are the root mean square error of the training, cross validation, and test sets, respectively. Models were built using 1000 training and 1000 test concentrations and samples

Thiol	*R* ^2^	*Q* ^2^ (CV)	*Q* ^2^ (test)	RMSEC (ppm)	RMSECV (ppm)	RMSEP (ppm)
Results from PLS-1 models						
3M3MH	0.9807	0.9795	0.9826	0.0385	0.0393	0.0359
2M3MB	0.9726	0.9704	0.9739	0.0459	0.0474	0.0438
3MH	0.9811	0.9684	0.9738	0.0379	0.0485	0.0439
2M3MP	0.9738	0.9733	0.9712	0.0445	0.0447	0.0459
Results from PLS-2 model						
3M3MH	0.9849	0.9774	0.9827	0.0341	0.0413	0.0359
2M3MB	0.9827	0.9689	0.9754	0.0365	0.0485	0.0428
3MH	0.9808	0.9639	0.9740	0.0383	0.0519	0.0436
2M3MP	0.9807	0.9711	0.9713	0.0382	0.0464	0.0458

Alongside PLS-R, multivariate curve resolution-alternating least squares (MCR-ALS) was another multivariate technique used to detect and quantify the four thiols in a mixture. Initially, MCR-ALS was applied to spectra which were only normalised using standard normal variate (SNV) normalisation. However, this led to good resolution for only one of the thiols, (3M3MH), in which there was a high consistency between the resolved spectrum and the actual reference SERS spectrum. In particular, there was poor resolution for 2M3MB and 2M3MP. This resulted in very different resolved spectra compared to the actual spectra of the thiols. This is likely because there are a high number of overlapping peaks between the spectra of the two thiols. Consequently, the spectral profiles were poorly resolved and there was poor correlation between the predicted and known concentrations using MCR-ALS. However, when the first derivative of the pure thiol spectra and the spectra of the mixtures was used, the resolved spectra of all four thiols showed better consistency with the corresponding actual spectra (Fig. S17†). 3M3MH and 2M3MP showed the best resolution and the best correlation between predicted and known concentrations (*R*^2^ = 0.9071 and 0.906 respectively) (Fig. S17A†). In both first derivative spectra for 3M3MH and 2M3MP, there are a high number of unique peaks in both spectra which are easier to resolve in contrast to 3MH and 2M3MB which have some overlapping peaks. As a result, the concentration profiles of 3M3MH and 2M3MP were easier to resolve than 2M3MB and 3MH. Nevertheless, the results of quantification of MCR-ALS were much poorer than those of the PLS-R models, even for the best resolved thiols, indicating that well resolved spectra could not guarantee a good correlation between resolved concentration profiles and the corresponding known concentrations.

### Blind test measurements of multiple thiols in a mixture

**Fig. 5:**
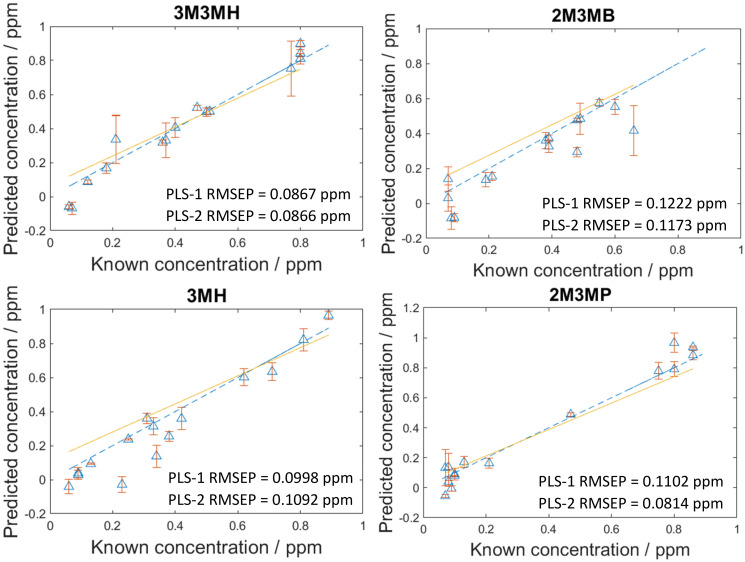
PLS-2 prediction plots of the blind test data for each of the thiols of interest in a multiplex mixture. RMSEP is the root mean square error of the test set.

As the concentration of thiols could be predicted with known concentrations using PLS-R, a blind test experiment was performed to test whether these models could be used to predict the concentration of thiols at unknown concentrations in multiplexed mixtures of all four thiols. This involved one person (C.L.) generating the mixtures at concentrations only known to them and another person (A.C.) measuring and analysing the data. In this process, 15 new mixtures were generated and analysed two weeks after the initial 120 mixtures. To analyse the data, both PLS-1 and PLS-2 models were generated with all 120 mixtures as the training set and the 15 new mixtures were used as the test set. The number of latent variables used to produce the original PLS-R models for the 120 mixtures were used for these new models to keep the calculations the same and the results comparable. As seen in Fig. 5, the RMSEP values between the PLS-1 and PLS-2 models show similar agreement to one another. 3M3MH shows the best agreement between models with PLS-1 and PLS-2 RMSEP values of 0.0867 pm and 0.0866 ppm respectively. Additionally, the amount of error for the models is still low, with all RMSEP values less than 0.123 ppm. However, the RMSEP values of these prediction models are higher in value than the RMSEP values for the initial 120 mixtures. This may be due to day-to-day variations from performing the experiment two weeks after the initial experiments with the 120 mixtures. Nevertheless, these results show that by using SERS and PLS-R models, these malodorous thiols can be detected and quantified simultaneously in a mixture. By performing a blind test, this demonstrates the reproducibility and reliability of the original PLS-R models used to predict the concentrations of thiols present in the mixtures.

The robustness and reliability of these PLS-R models could have been further proven by performing a double-blind study with mixtures produced externally so none were aware of the concentrations present in the mixtures. This would have helped to improve the validity in these methods. Furthermore, these thiols are produced biologically by the microbiome, so further investigation could involve examining the thiol mixtures in biological models that represent what is generated by the skin microbiome. This would further show the real-life usability and application of SERS for measuring malodorous thiols. However, despite not doing this, this is one of the few SERS quantification studies where samples with concentrations unknown to the analyst have been assessed.

## Conclusion

We have demonstrated that four malodorous thiols: 3MH, 3M3MH, 2M3MP and 2M3MB can be detected and quantified using optimised parameters for SERS with hydroxylamine-stabilised silver nanoparticles in solution. The LoD of the thiols were measured individually using both univariate and multivariate methods of analysis. The values of LoD found with PLS-R agreed with the range of LoDs found using univariate analysis on different peaks for each thiol and using PLS-R. The LoD of each thiol was calculated to be between 0.0114 ppm and 0.5004 ppm for univariate analysis and between 0.0153 ppm and 0.0227 ppm using PLS-R. Moreover, using PLS-R with bootstrapping validation, the thiols could be quantified in 120 mixtures containing different concentrations of each of the four thiols. PLS-1 and PLS-2 models were both produced to model and quantify the thiols in a mixture. The models produced very similar results for error and goodness-of-fit for both the training and test data. Furthermore, the values for *Q*^2^ of the test data were greater than 0.96 and RMSEP values were less than 0.046 ppm for both models. Therefore, both models showed good ability to predict the concentrations of thiols in a mixture. To validate this further, these models were used for 15 new mixtures with analytes at unknown concentrations. Both models showed low values for RMSEP of less than 0.1222 ppm with this data as the test set. This demonstrates that malodorous thiols in a mixture can be identified and quantified using SERS and chemometrics. These results demonstrate the usability of SERS for detecting and quantifying analytes in multiplex solutions, including analytes which may have very similar spectra to one another. Additionally, SERS can be used with chemometrics to predict unknown concentrations of analytes in multiplex solutions simultaneously. These methods could be used in other areas of research where SERS is being tested as a potential technique to identify and measure multiple analytes. This is one a few studies to display the quantitative multiplex capabilities of SERS and to validate the methods used using unknown concentrations.

The advantages to using SERS for detecting these malodorous thiols are that the thiols will chemisorb to the metal nanostructured surface and there are portable Raman devices. This means that the Raman signal of the thiols is significantly enhanced using SERS and real time analysis can be performed to measure the malodorous thiols produced from the axilla. To achieve this, future research will focus on developing a solid SERS substrate which can detect these gaseous thiols. This should reduce data acquisition time and, therefore, more information may be obtained about the thiols. However, there are challenges which need to be considered for future development of using SERS for the detection of these malodorous thiols. Primarily, it has been found that more volatile compounds have been identified from axillary sweat compared to urine and saliva. This makes the samples very complex, especially as volatile profiles of the axilla vary between individuals.^1,75^ These volatiles may be present at higher concentrations than the thiols of interest. Furthermore, as these thiols are highly volatile, there may still be difficulties in preventing the loss of thiols before they are measured with a portable Raman spectrometer. Therefore, targeted approaches, such as methods for trapping the gases to the surface of a SERS substrate, using more complex supervised models for identifying the thiols or using reporter molecules which are specific to the thiols would be needed for SERS detection of malodorous thiols from the axilla.

Nevertheless, overall, these results display promise for future work in developing SERS for measuring the thiols in a gaseous state and for future development and use for SERS in real-life application to quantify malodorous thiols produced from the axilla.

## Author contributions

A.C. contributed towards experimental design, investigation, data analysis, data interpretation and writing the original draft. C.L. contributed towards experimental design, investigation, and data interpretation. Y.X. contributed towards experimental design, data analysis and data interpretation. A.M. and S.M. contributed towards conceptualisation, experimental design, and supervision. R.G. contributed towards conceptualisation, experimental design, data interpretation and supervision. All authors contributed towards reviewing and editing the writing.

## Data availability

Data processing algorithms are available *via*: https://github.com/Biospec/.

## Conflicts of interest

There are no conflicts to declare.

## Supplementary Material

AN-149-D4AN00762J-s001
