# Supplementary material for: Using surface-enhanced Raman scattering for simultaneous multiplex detection and quantification of thiols associated to axillary malodour
Source: Analyst. 2024 Jun 24;149(15):3989–4001. doi: 10.1039/d4an00762j (PMC11262063; doi:10.1039/d4an00762j)
Supplement: AN-149-D4AN00762J-s001 [file AN-149-D4AN00762J-s001.pdf]

Electronic Supplementary Material (ESI) for Analyst.
This journal is © The Royal Society of Chemistry 2024

Supplementary Information 1

Using surface-enhanced Raman scattering for simultaneous multiplex detection and quantification of thiols associated to underarm odour

Amy Colleran, Cassio Lima, Yun Xu, Allen Millichope, Stephanie Murray and Royston Goodacre

Methods

Nanoparticle Characterisation

UV-Vis spectroscopy was used to determine the surface plasmon resonance band λ_{max} of the different nanoparticles. 2 mL nanoparticle suspension was pipetted into a quartz pipette (path length= 10 mm) and inserted into a sample holder of Jenway 7200 scanning spectrophotometer (Cole-Parmer, Staffordshire, UK). Absorbance spectra was obtained over a wavelength range of 300-800 nm. Hydroxylamine-stabilised silver nanoparticles were diluted in a 1:4 ratio in water and citrate-stabilised gold nanoparticles were diluted in a 1:3 ratio in water. Scanning electron microscopy (SEM) was used examine the morphology of the nanoparticles. The nanoparticles were deposited on to Si wafers and imaged using a Hitachi S-4800 SEM. The hydrodynamic diameter and zeta-potential of the nanoparticles were measured using the Malvern Zetasizer Nano ZS (Malvern Panalytical, Malvern, UK) fitted with a 532 nm laser.

SERS optimisation experiments

As these thiols have not previously been analysed using SERS, it is important to optimise the SERS parameters to ensure reproducible results with the greatest SERS enhancement. To do this, all parameters were optimised for each different set of nanoparticles using (3MH) as the analyte of choice. 3MH was chosen due to its commercial availability. Parameters which were optimised and investigated were: excitation wavelength, analyte concentration, colloid volume, aggregating agent, aggregating agent concentration, pH and aggregation time in this order. Once one parameter was optimised, the optimised condition would then be used in further optimisation experiments. When optimising the colloid volume, the total volume of the SERS mixture remained constant. Once the optimised parameters were determined for each type of nanoparticle, these parameters were tested with 3M3MH, 2M3MB and 2M3MP as the analytes of interest. 10,000 ppm stock solutions of 3M3MH, 3MH, 2M3MB and 2M3MP were prepared by dissolving each thiol in 50 % acetonitrile solution. All other concentrations of thiols were prepared using these stock solutions. The final parameters chosen for all other experiments were based on a compromise between all four analytes.

To keep all future measurements consistent between thiols, all stock solutions of thiols were dissolved in 50% acetonitrile. At low concentrations, peaks that can be assigned to acetonitrile could not be seen by eye and the spectra of the thiols dominated instead.

To test the reproducibility and stability of the SERS enhancement from measuring thiols with one batch of hAgNPs, four more batches of hAgNPs were produced. Measurements were taken of 3M3MH, 2M3MB, 3MH and 2M3MP at three concentrations between 3 ppm and 0.0058 ppm, each at a high, middle, and low concentration within that range. All batches of nanoparticles were tested with each thiol at each concentration.

To determine the most appropriate concentration to measure thiols in a multiplex solution so that the concentration did not surpass one monolayer coverage of nanoparticles, the six combinations of two thiols in a mixture were measured at equal concentration in solution. The total concentrations of two thiols in a mixture were between 0.404 ppm and 100 ppm.

Changes in the spectrum of a mixture of thiols were initially examined by keeping three thiol thiols at a constant concentration of 0.75 ppm. The other thiol was then added into separate aliquots of the mixture at concentrations of 0.2 ppm, 0.5 ppm, 1.2 ppm, 1.5 ppm and 2 ppm. This experiment was repeated for the remaining three thiols.

Nanoparticle Characterisation

Citrate-stabilised gold nanoparticles (cAuNPs), citrate-stabilised silver nanoparticles (cAgNPs) and hydroxylamine-stabilised silver nanoparticles (hAgNPs) were synthesised for SERS measurements of 3-mercapto-hexan-1-ol (3MH) dissolved in 20 % acetonitrile. To establish the surface plasmon band (λ_{max}) of the nanoparticles, the nanoparticles were measured using UV-Vis spectrometry. UV-Vis can also show the size distribution of the nanoparticles and a narrower peak indicates a more monodisperse colloid. [1] cAuNPs and cAgNPs showed absorption maximums at 527 nm and 436 nm respectively. cAuNPs show a wavelength in agreement with the literature.[2] However, the wavelength value for cAgNPs is longer than previously mentioned.[3] Furthermore, cAgNPs show a secondary peak which implies, alongside the broad peak, that there is a wide size distribution range of cAgNPs in solution (**Figure S1**). This is probably due to the method of synthesis. The Lee-Meisel method for synthesising silver nanoparticles does not produce nanoparticles which show the same morphology. This can lead to poor reproducibility and can lead to the broadening of the surface plasmon peak. [4] This can be seen from the SEM images of the cAgNPs which show a variety of shapes including rods, spherical nanoparticles and non-spherical nanoparticles (**Figure S2**). hAgNPs batches 1 to 5 all showed plasmon absorbances between 409 and 425 nm which is shorter than previously mentioned in the literature.[3]

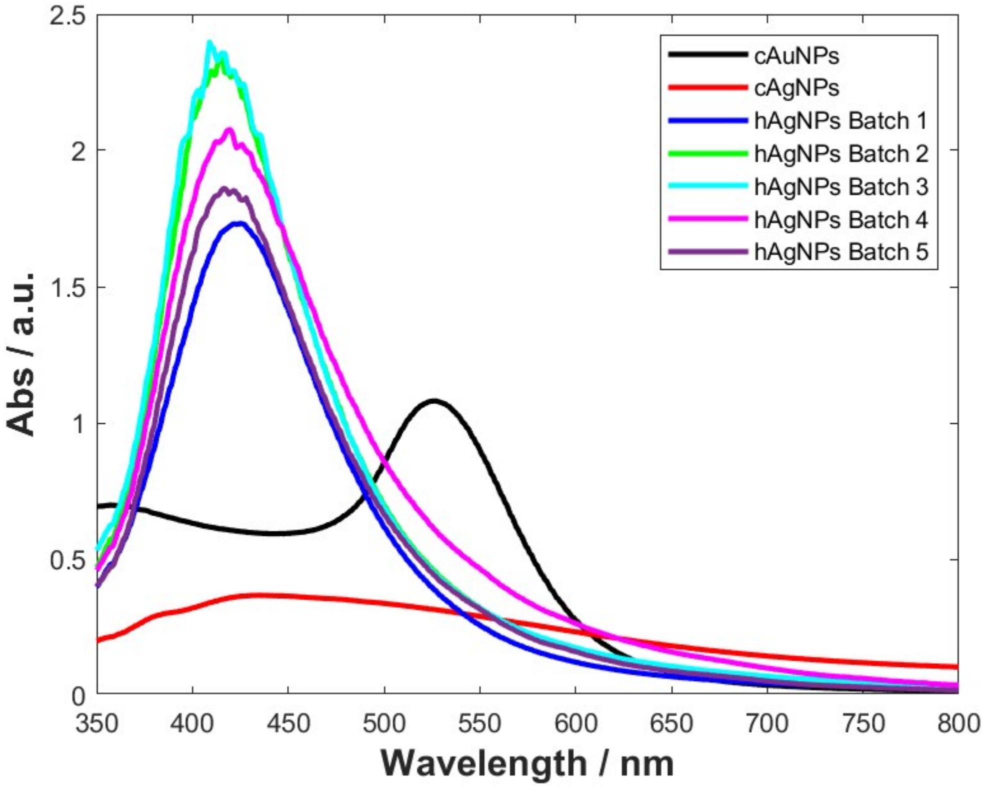

Figure S1: UV-Visible absorption spectra of citrate-stabilised gold nanoparticles (cAuNPs), citrate-stabilised silver nanoparticles (cAgNPs) and hydroxylamine-stabilised silver nanoparticles (hAgNPs).

To further characterise the nanoparticles, dynamic light scattering (DLS) was used to measure the hydrodynamic diameter, zeta potential and size distribution of the nanoparticles. The results of these measurements are displayed in **Figure S3** and **Figure S4**. cAuNPs and cAgNPs have hydrodynamic diameters of 27.82 ± 0.14 nm and 67.35 ± 0.53 nm respectively. hAgNPs batches 1 to 5 appeared to have diameters between 53.87 ± 0.45 nm and 74.03 ± 0.42 nm (**Figure S3**). hAgNPs batch 1 are larger in diameter than batches 2 to 5. This is because batch 1 was made at a different time to batches 2 to 5 as batches 2 to 5 were produced to test the stability and examine the reproducibility of the SERS signal using hAgNPs. Batch 1 was used for all measurements discussed in this research. All the nanoparticles produced showed some polydispersity in the size distribution as all polydispersity indexes were greater than 0.19. Moreover, smaller nanoparticles are clearly present in some solutions such as in cAuNPs and cAgNPs which further indicates that there is a wider size distribution of nanoparticles (**Figure S3).** Measuring the zeta-potential of the nanoparticles can show how stable the nanoparticles are in solution. For gold and silver nanoparticles, a potential value of -30 mV or less indicates that the nanoparticles are stable in solution [5]. All batches and types of nanoparticles showed a zeta-potential value between -38 mV and -44 mV which means that all nanoparticles produced were stable in solution.

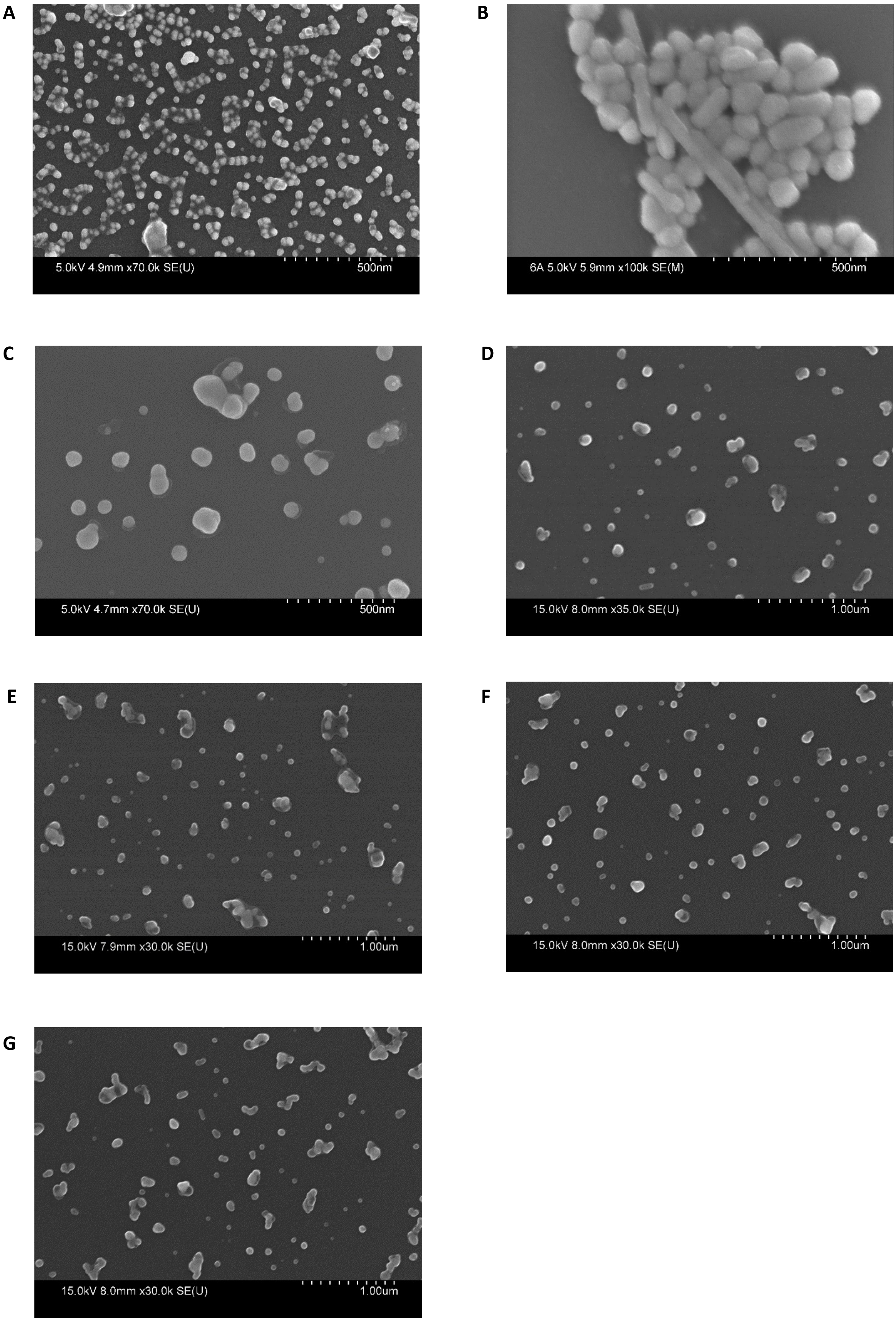

Figure S2: SEM images of **A.** cAuNPs, **B.** cAgNPs, **C.** hAgNPs batch 1, **D.** hAgNPs batch 2, **E.** hAgNPs batch 3, **F.** hAgNPs batch 4 and **G.** hAgNPs batch 5.

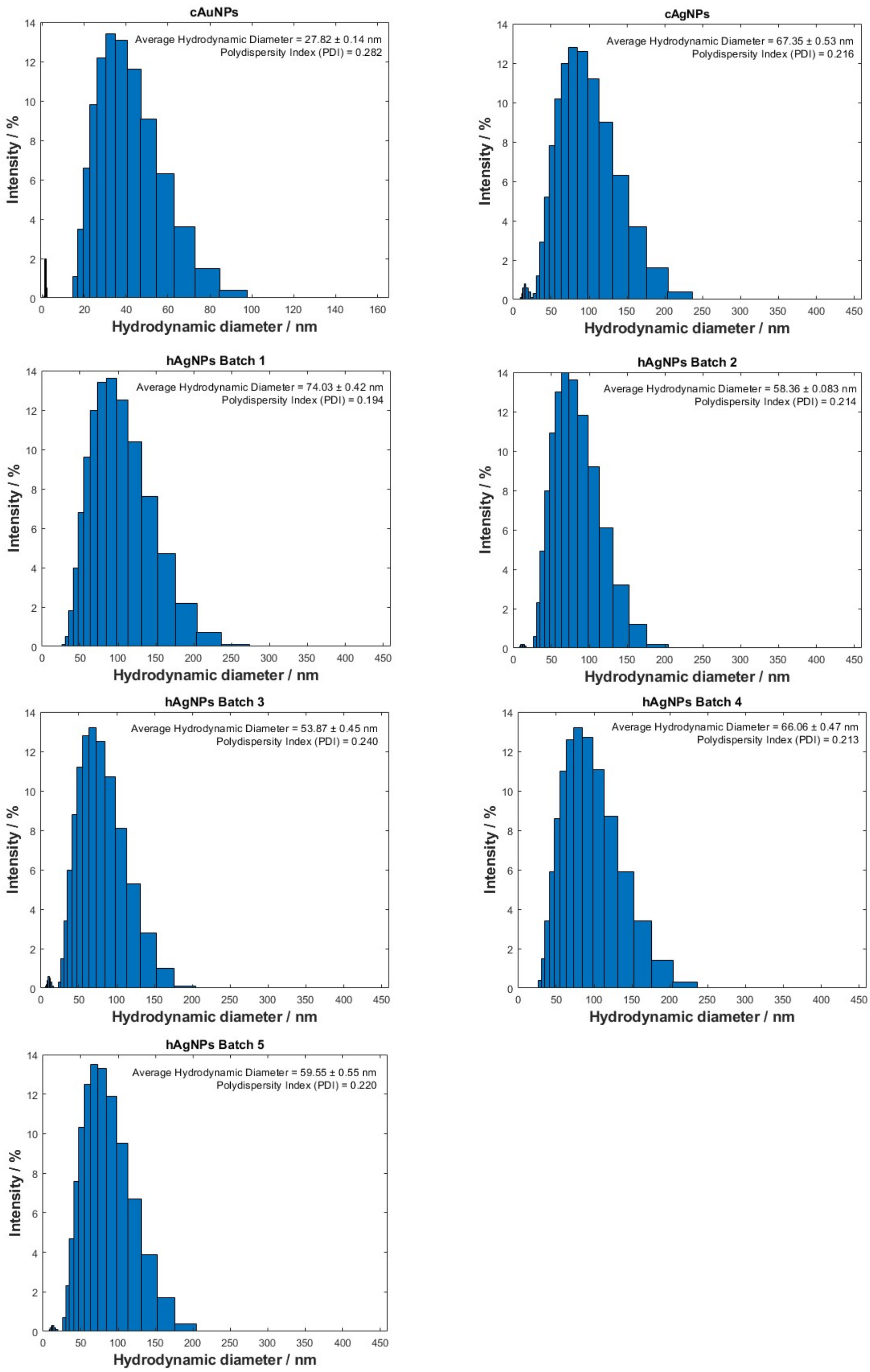

Figure S3: Results from the zetasizer of the average hydrodynamic diameter (n = 5) of citrate stabilised gold nanoparticles (cAuNPs), citrate stabilised silver nanoparticles (cAgNPs) and different batches of hydroxylamine stabilised silver nanoparticles (hAgNPs). Histograms display size distribution from intensity.

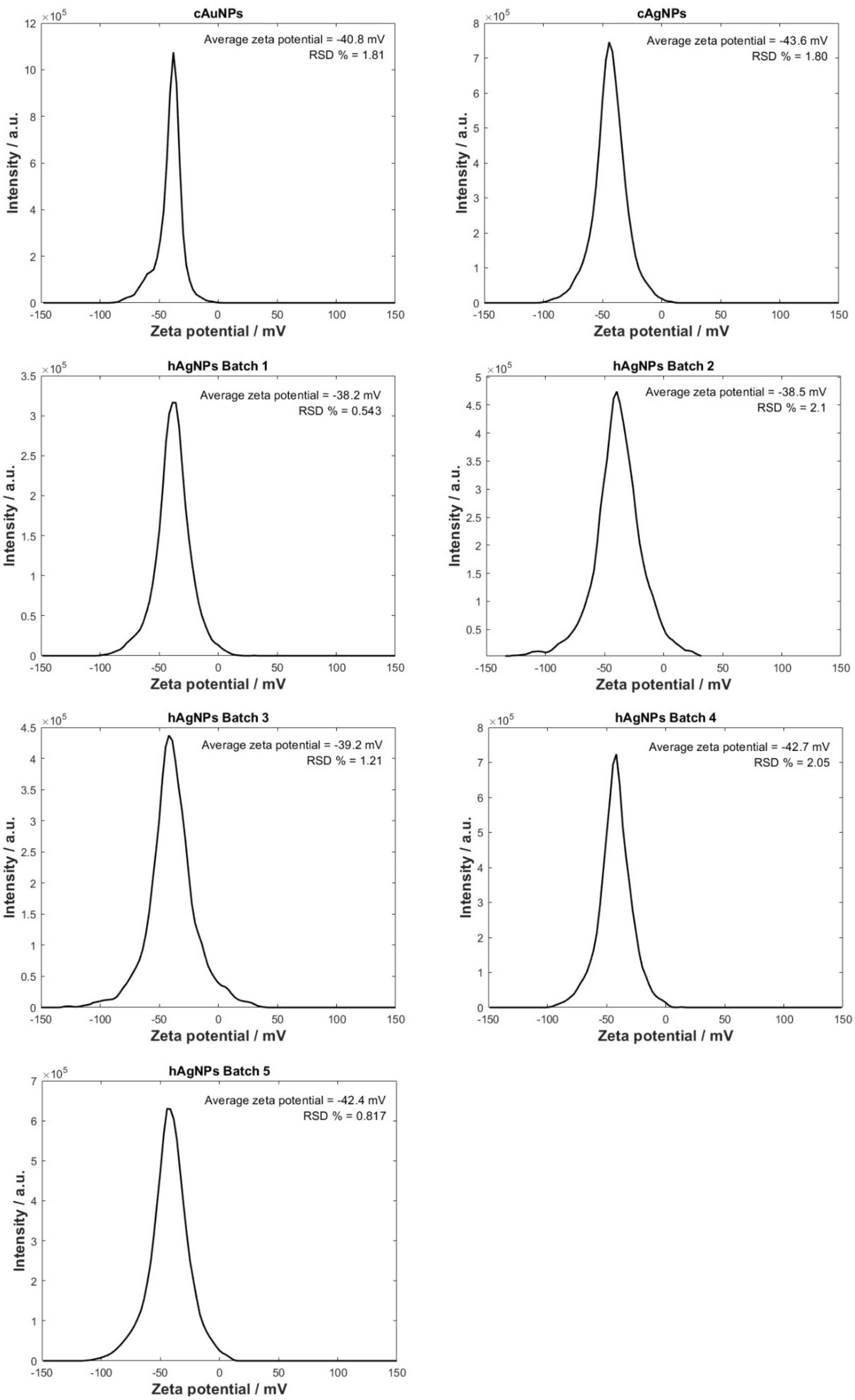

Figure S4: Results from the zetasizer of the average zeta potential (n = 5) of citrate stabilised gold nanoparticles (cAuNPs), citrate stabilised silver nanoparticles (cAgNPs) and different batches of hydroxylamine stabilised silver nanoparticles (hAgNPs).

SERS optimisation of thiols in solution

To decide on the optimal SERS parameters to measure the thiols, all parameters were optimised for each set of nanoparticles using 3-mercapto-hexan-1-ol (3MH) dissolved to a concentration of 10,000 ppm in 20% acetonitrile as the analyte. The parameters optimised for each set of nanoparticles were: excitation wavelength (633 nm and 785 nm spectrometers), aggregating agent (NaCl, KCl, $MgSO_4$, Na_2SO_4, $(NH_4)_2SO_4$, K_2SO_4, KNO_3 and poly-L-lysine (0.01 %)), aggregating agent concentration (0.1 M to 1 M in 0.1M increments), pH (between pH 1 and pH 14), volume ratio of nanoparticles to analyte (10% volume of nanoparticles to 90% volume of nanoparticles in 10% increments) and aggregation time (measured over 60 min in 30 s increments). 3 replicate samples were made for each measurement. For each parameter, the condition that showed the greatest SERS enhancement for 3MH and the most reproducible spectrum of 3MH was chosen. This parameter was then kept the same whilst other parameters were optimised. For hAgNPs, there appeared to be a change in colour of the solution from milky grey-orange to a darker grey-orange after the addition of 3MH without any aggregating agent. This indicates that 3MH caused aggregation of the nanoparticles. However, the SERS enhancement was not as big as when an aggregating agent is used to measure 3MH. Therefore, SERS parameters were also optimised for hAgNPs without any aggregating agent. After the parameters had been optimised for each type of nanoparticles (**Table 1**, **Tables S1 – S3**), these parameters were tested on 3-methyl-3-mercapto-hexan-1-ol (3M3MH), 2-methyl-3-mercapto-butan-1-ol (2M3MB) and 3-methyl-3-mercapto-pentan-1-ol (2M3MP). These thiols were made to a concentration of 10,000 ppm in 50 % acetonitrile as the thiols would not dissolve in a lower percentage of acetonitrile in solution. For future experimental work, 3MH was also dissolved to 10,000 ppm in 50% acetonitrile so that the amount of acetonitrile present did not vary between analytes.

When comparing the spectra for using different nanoparticles for each analyte, the nanoparticles which showed the greatest enhancement across all spectra were hAgNPs using an aggregating agent (NaCl (0.2 M)) (**Figure S5**). For the other analytes, no change in colour of solution was seen when using hAgNPs with no aggregating. This could suggest that 3MH has different chemical properties compared to the other thiols. Alternatively, that the conditions used would have to be modified to see a greater enhancement without an aggregating agent. This could be done by increasing the pH to increase the number of anionic thiol compounds in solution. Using this approach, this would improve the binding to the silver surface. Another method to improve the enhancement without an aggregating agent would be to increase the volume of nanoparticles. It may also be due to the higher percentage of acetonitrile in the solution which may be surrounding the nanoparticles and preventing interaction between the nanoparticles and analyte. As hAgNPs showed good enhancement with an aggregating agent for all thiols, the standard method selected for this study was to make the thiols to the desired concentration in pH 11 solution followed by transferring 130 µL of analyte solution to 270 µL of hAgNPs. 50 µL of NaCl (0.2 M) was then added to the mixture and the spectrum was acquired immediately with an acquisition time of 30 s with a 785 nm Raman spectrometer.

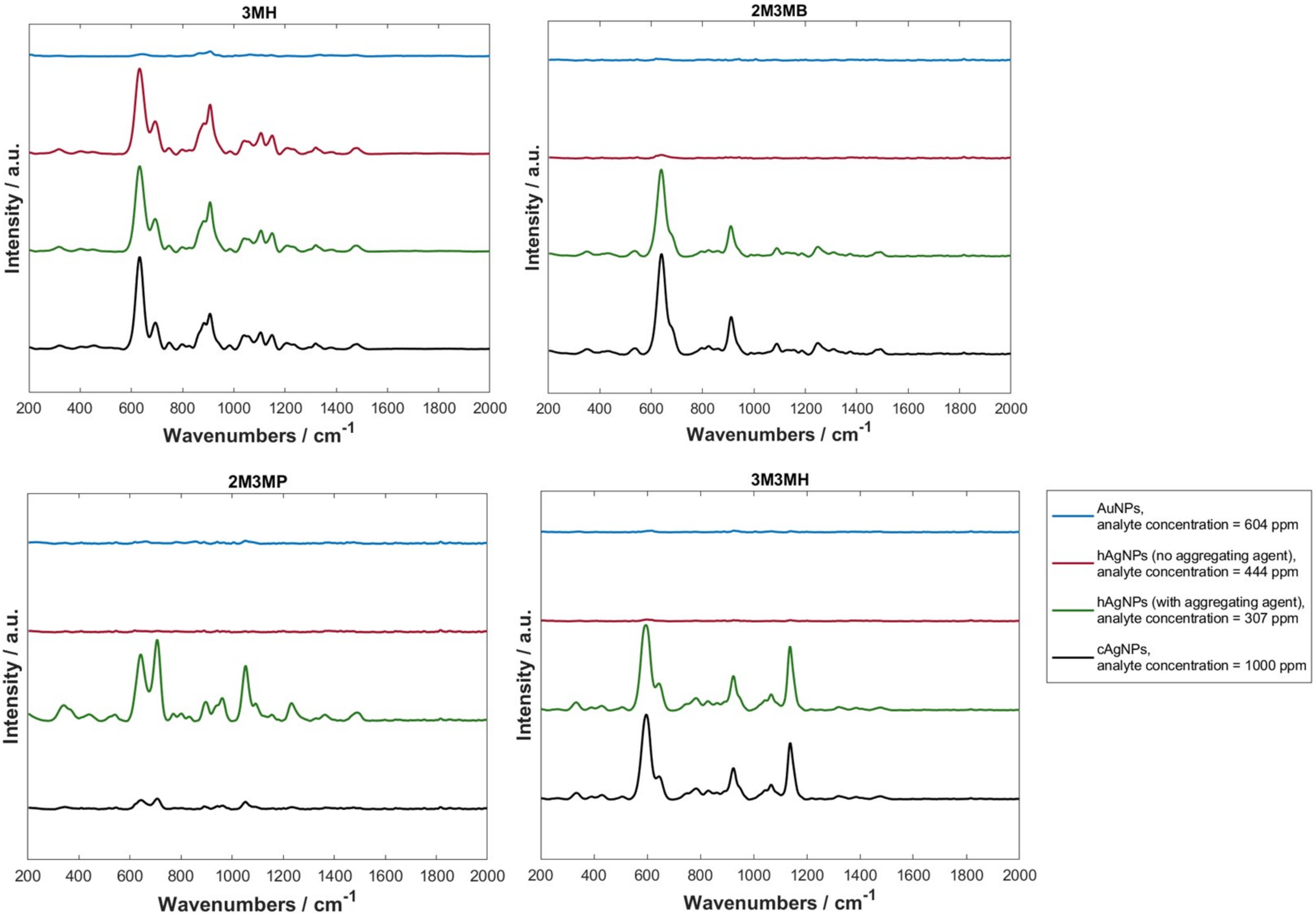

Figure S5: Average SERS spectra of 3M2MP, 3M2MB, 3M3MH and 3MH in 50 % acetonitrile solution using optimised conditions for citrate-stabilised gold nanoparticles (AuNPs), hydroxylamine-stabilised silver nanoparticles (hAgNPs) with no aggregating agent and with an aggregating agent (NaCl, 0.2M) and citrate-stabilised silver nanoparticles (cAgNPs). The spectra have been baseline corrected, smoothed and vector normalised.

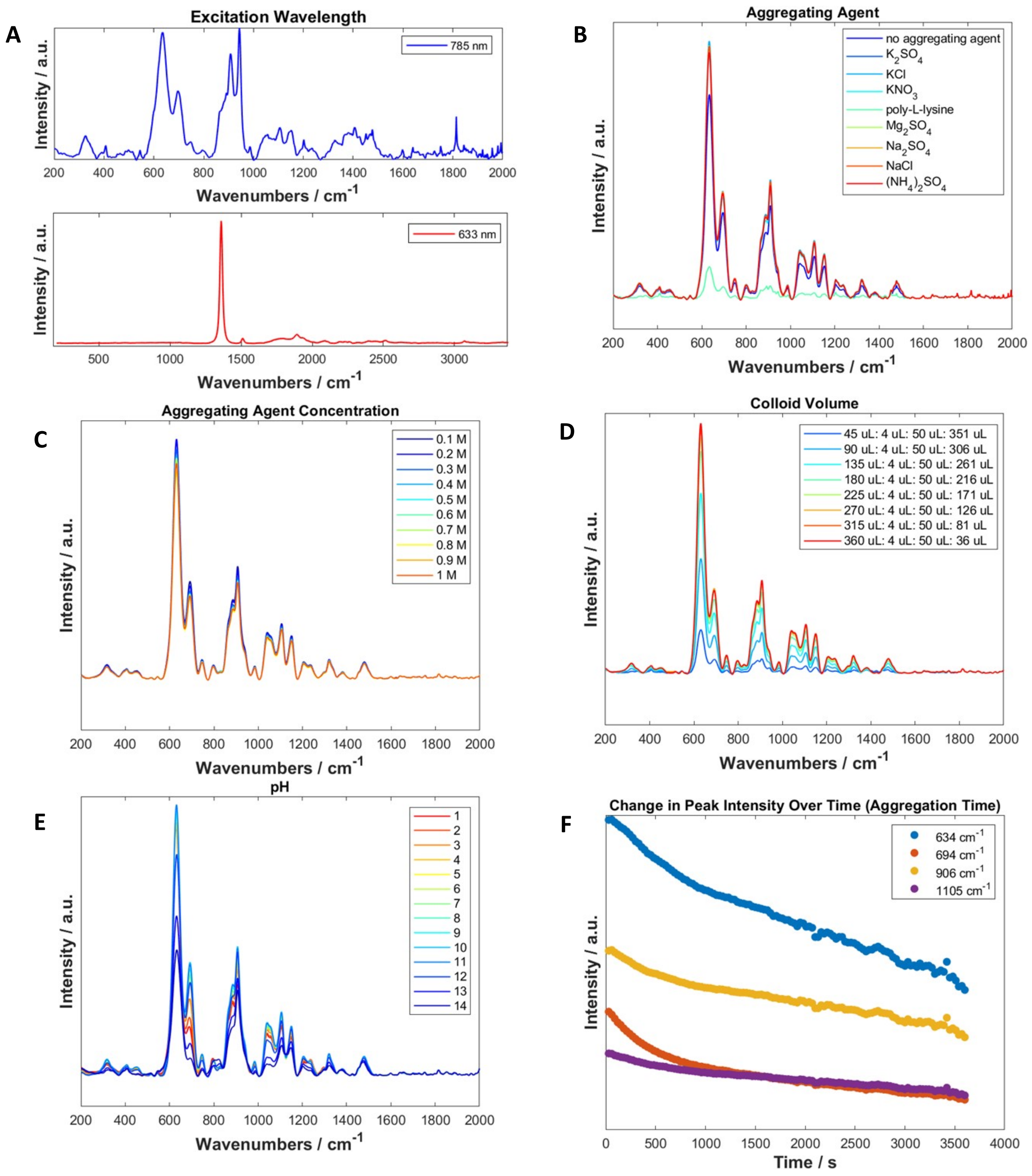

Figure S6: Parameters optimised for SERS with hydroxylamine-stabilised silver nanoparticles (hAgNPs) with an aggregating agent. **A.** Comparison of spectra of 3MH measured at 785 nm and 633 nm. **B.** Comparison of SERS measurements of 3MH using different aggregating agents. **C.** Comparison of SERS measurements of 3MH using different aggregating agent concentrations. **D.** Comparison of SERS measurements of 3MH using different colloid volumes. The ratios represent the volumes of hAgNPs: 3MH: NaCl: water in the mixture. **E.** Comparison of SERS measurements of 3MH using different pHs. **F.** Changes in peak intensity of various peaks in 3MH Raman spectrum over time with a measurement taken every 30 s to identify a suitable aggregation time. All spectra were baseline corrected only.

Table S1: Optimised SERS parameters used for measuring thiols with citrate-stabilised gold nanoparticles (cAuNPs).

**Excitation wavelength**	785 nm
**Acquisition time**	30 s
**Nanoparticles**	cAuNPs
**Aggregating agent**	$MgSO_4$
**Aggregating agent concentration**	0.5 M
**Volume ratio**<br>**(cAuNPs : analyte : aggregating agent)**	135 µL : 265 µL : 50 µL
**pH**	6
**Aggregation time**	3 min

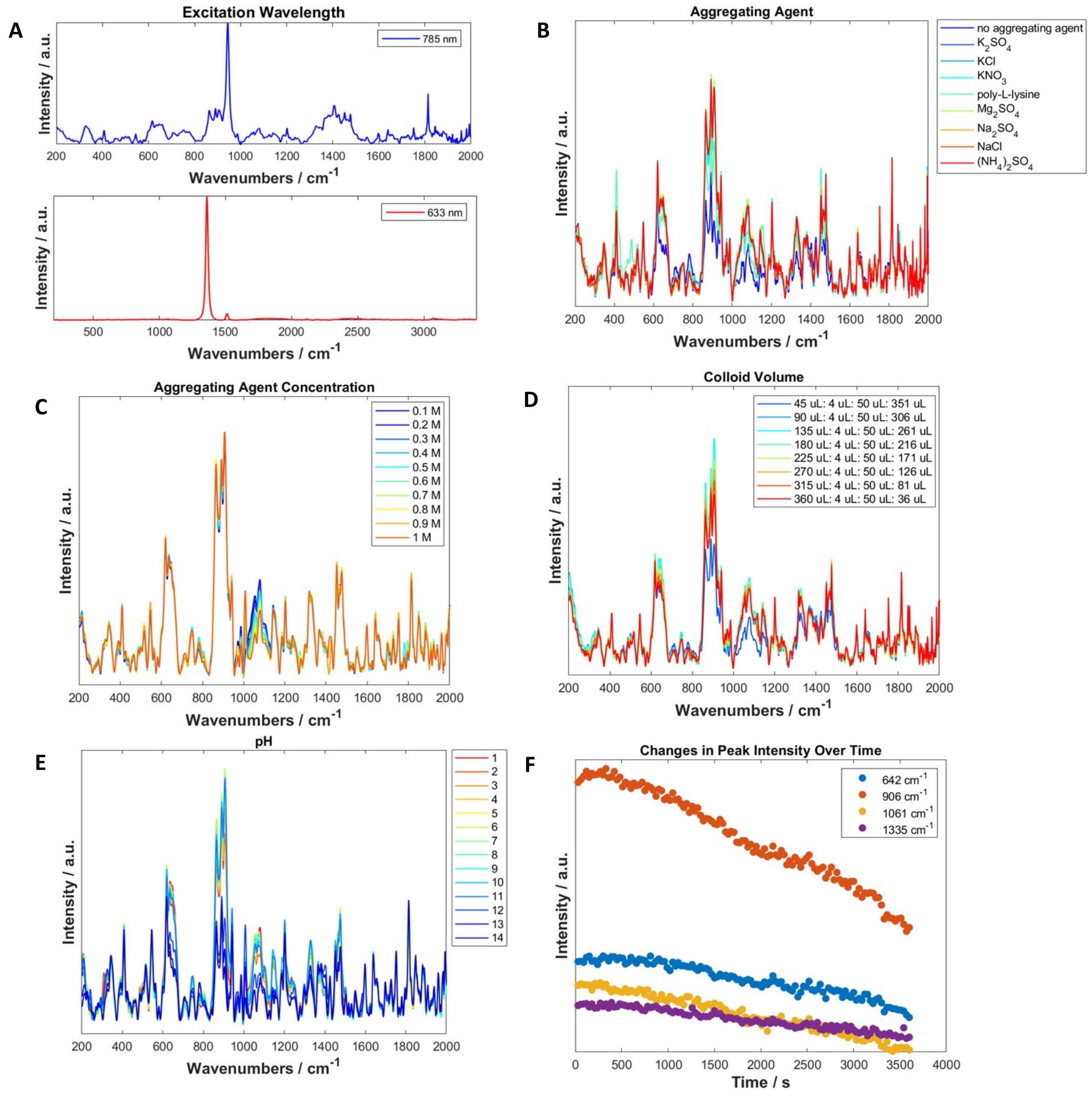

Figure S7: Parameters optimised for SERS with citrate-stabilised gold nanoparticles (cAuNPs). **A.** Comparison of spectra of 3MH measured at 785 nm and 633 nm. **B.** Comparison of SERS measurements of 3MH using different aggregating agents. **C.** Comparison of SERS measurements of 3MH using different aggregating agent concentrations. **D.** Comparison of SERS measurements of 3MH using different colloid volumes. The ratios represent the volumes of hAgNPs: 3MH: NaCl: water in the mixture. **E.** Comparison of SERS measurements of 3MH using different pHs. **F.** Changes in peak intensity of various peaks in 3MH Raman spectrum over time with a measurement taken every 30 s to identify a suitable aggregation time. All spectra were baseline corrected only.

Table S2: Optimised SERS parameters used for measuring thiols with citrate-stabilised silver nanoparticles (cAgNPs).

**Excitation wavelength**	785 nm
**Acquisition time**	30 s
**Nanoparticles**	cAgNPs
**Aggregating agent**	NaCl
**Aggregating agent concentration**	0.1 M
**Volume ratio (cAgNPs : analyte : aggregating agent)**	360 µL : 40 µL : 50 µL
**pH**	7
**Aggregation time**	5 min

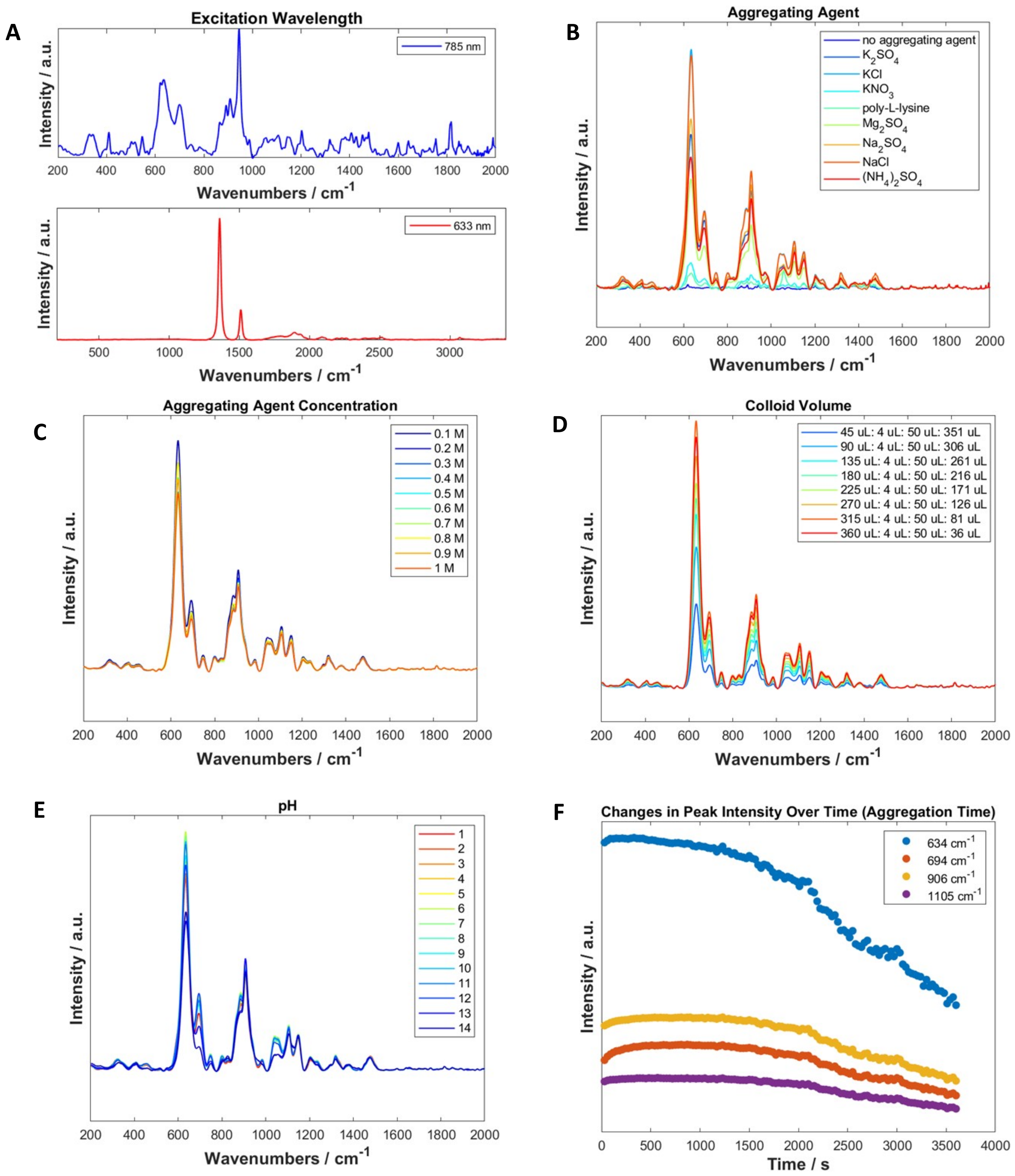

Figure S8: Parameters optimised for SERS with citrate-stabilised silver nanoparticles (cAgNPs). **A.** Comparison of spectra of 3MH measured at 785 nm and 633 nm. **B.** Comparison of SERS measurements of 3MH using different aggregating agents. **C.** Comparison of SERS measurements of 3MH using different aggregating agent concentrations. **D.** Comparison of SERS measurements of 3MH using different colloid volumes. The ratios represent the volumes of hAgNPs: 3MH: NaCl: water in the mixture. **E.** Comparison of SERS measurements of 3MH using different pHs. **F.** Changes in peak intensity of various peaks in 3MH Raman spectrum over time with a measurement taken every 30 s to identify a suitable aggregation time. All spectra were baseline corrected only.

Table S3: Optimised SERS parameters used for measuring thiols with hydroxylamine-stabilised silver nanoparticles (hAgNPs) without an aggregating agent.

**Excitation wavelength**	785 nm
**Acquisition time**	30 s
**Nanoparticles**	hAgNPs
**Volume ratio (hAgNPs : analyte)**	360 µL : 90 µL
**pH**	12
**Aggregation time (s)**	30 s

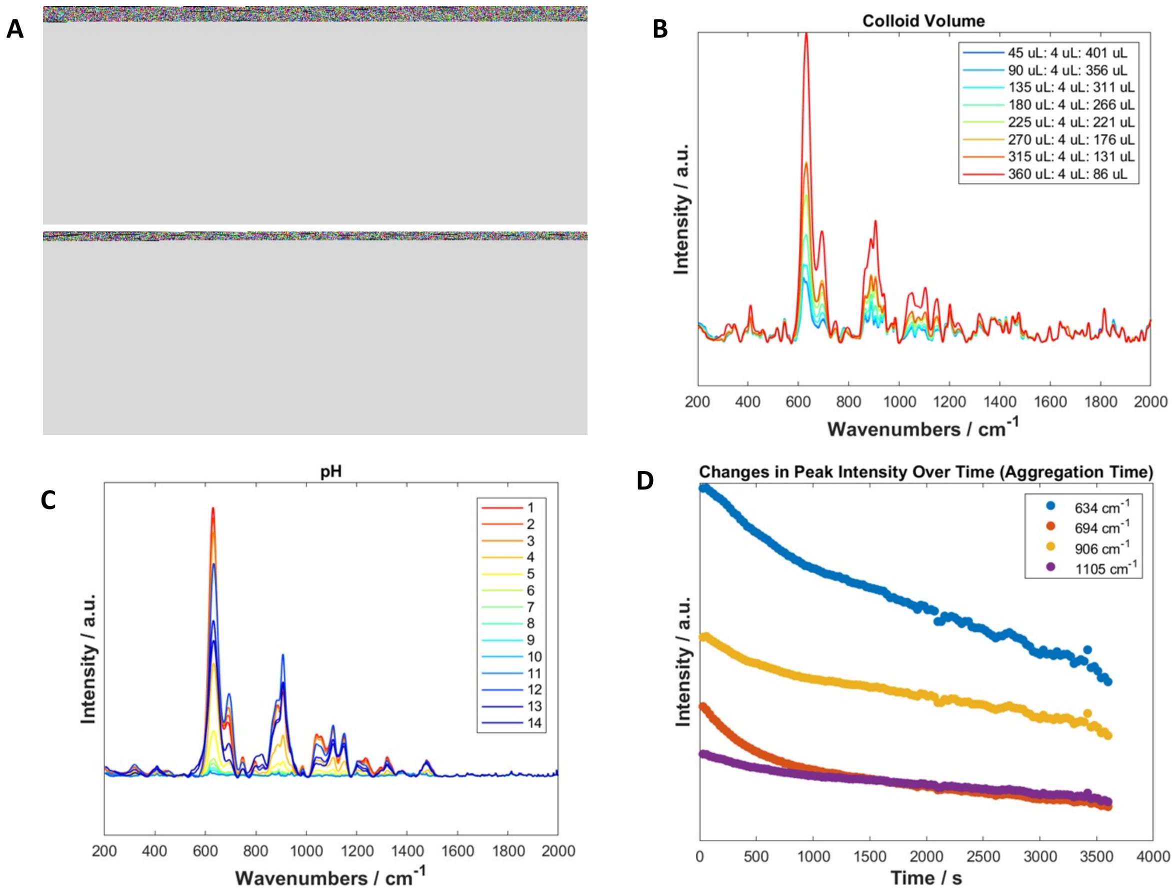

Figure S9: Parameters optimised for SERS with hydroxylamine-stabilised silver nanoparticles (hAgNPs) without an aggregating agent. **A.** Comparison of spectra of 3MH measured at 785 nm and 633 nm. **B.** Comparison of SERS measurements of 3MH using different colloid volumes. The ratios represent the volumes of hAgNPs: 3MH: NaCl: water in the mixture. **C.** Comparison of SERS measurements of 3MH using different pHs. **D.** Changes in peak intensity of various peaks in 3MH Raman spectrum over time with a measurement taken every 30 s to identify a suitable aggregation time. All spectra were baseline corrected only.

Reproducibility of the SERS enhancement using different batches of hAgNPs

As the key parameter to any SERS measurement is the nanoparticles or nanomaterials used, it is important to look at the reproducibility and stability of the SERS enhancement for the analyte of interest. This is done by testing different batches of the same SERS substrate with the analyte. Therefore, the four different thiols were measured at three concentrations using five batches of hAgNPs.

In general, the intensity of the thiol spectrum appeared to increase with increasing diameter of nanoparticles (**Figures S6-S9**). This is to be expected as it is known that SERS electromagnetic enhancement increases with increasing particle size.[6] However, these results were not seen for 2M3MB (**Figure S7**). For 2M3MB, batches 2 to 5 show greater enhancement of the 2M3MB spectrum compared to batch 1. In particular, at 1.04 ppm, the signal-to-noise ratio for batch 1 is much lower than for batches 2 to 5. It isn't clear why batch 1 shows a poorer enhancement. However, batches 2 to 5 were made on the same day which was later than batch 1. This was to make comparisons to the original batch, batch 1. This could be why there is a difference in size range between batch 1 and batches 2 to 5 and therefore could account for the variability in results. Overall, the trend remains the same between batches. As the concentration of the thiols decreases, there is a similar decrease in intensity of the spectrum between batches.

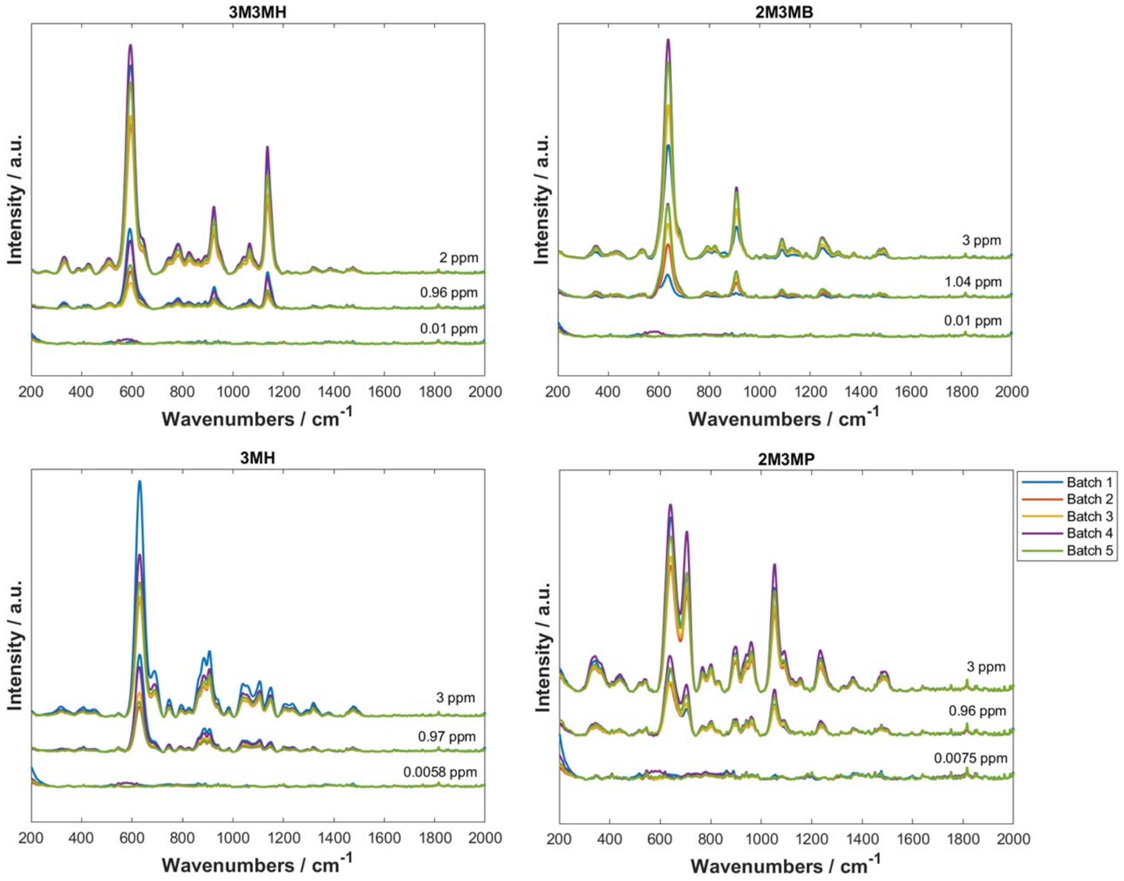

Figure S10: Comparison of spectrum of thiols at three different concentrations using five different batches of hAgNPs. Data has been baseline corrected only.

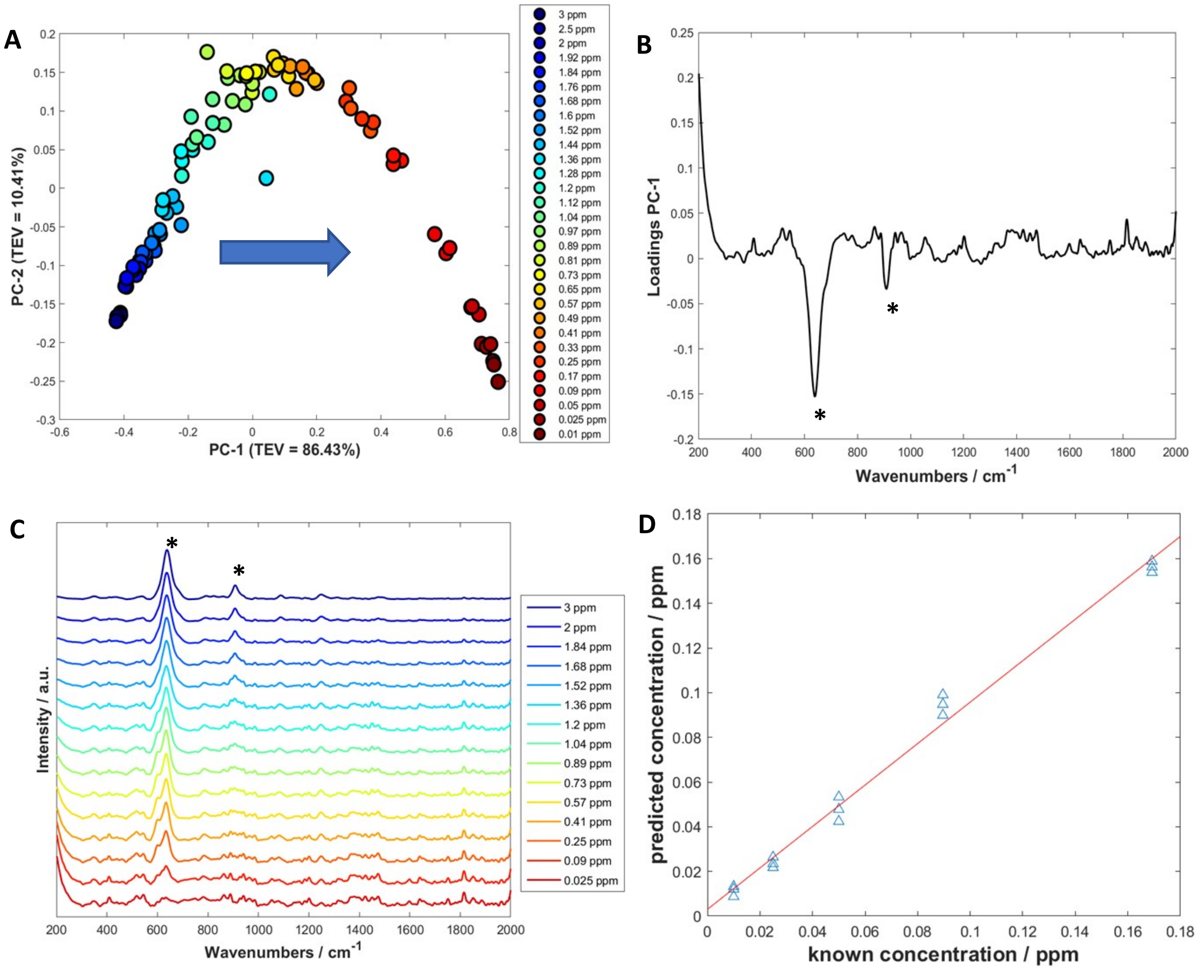

Figure S11: A. A PCA scores plot of 2M3MB at varying concentrations between 0.01 ppm and 3 ppm. The arrow indicates direction of decreasing concentration and TEV is the total explained variance. **B.** Loadings plot of PC-1. **C.** Representative spectra of 2M3MB varying in concentration between 0.01 ppm and 3 ppm (blue). **D.** PLS-R prediction plots of the test data for 2M3MB and used to calculate LoD using multivariate calculations. Asterisks in **B** and **C** represent the peaks chosen for the univariate calculation of the limit of detection (LoD). PLS-R models were produced using k-fold leave-one-out double cross validation. Data for figures **A – C** has been baseline corrected, smoothed and vector normalised.

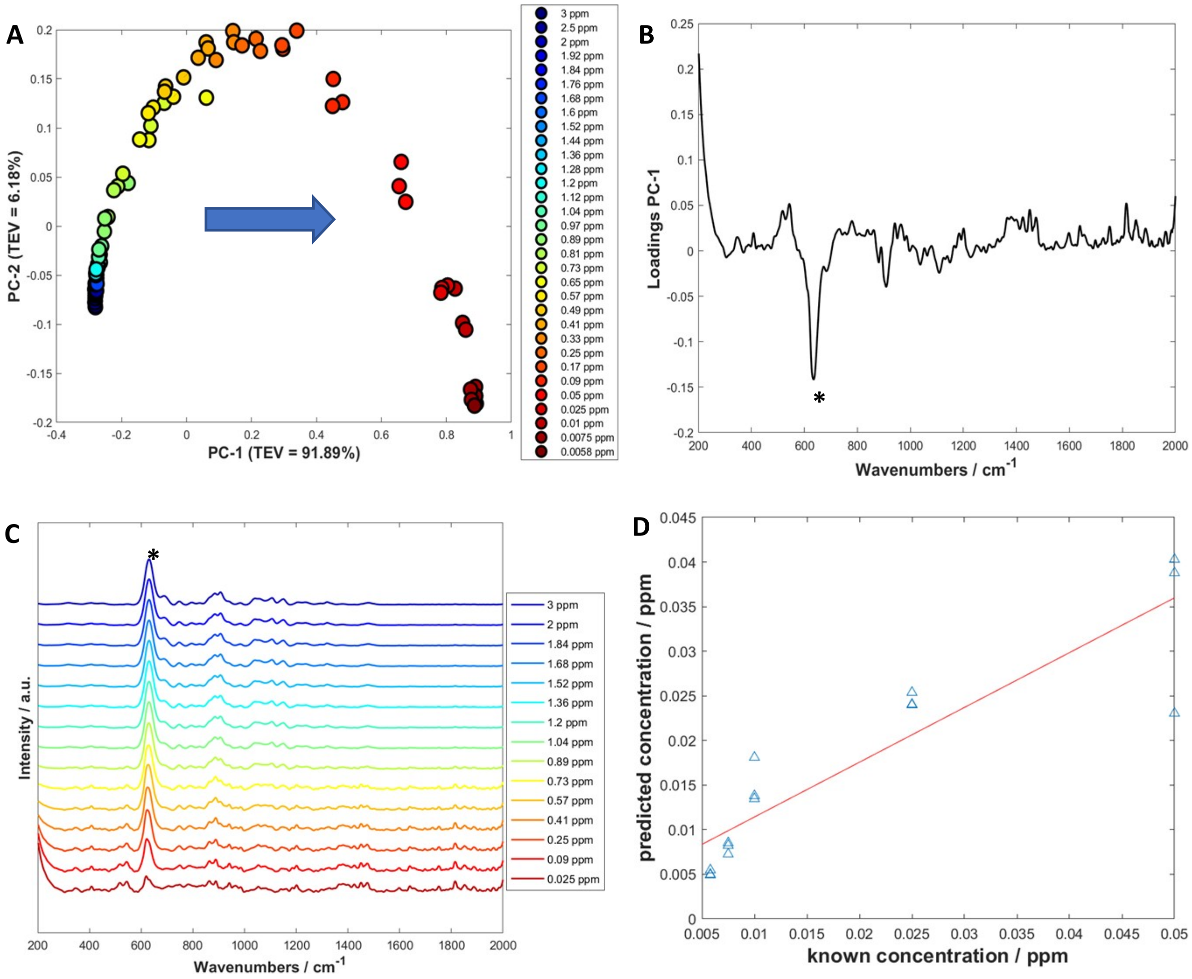

Figure S12: A. A PCA scores plot of 3MH at varying concentrations between 0.0058 ppm and 3 ppm. The arrow indicates direction of decreasing concentration and TEV is the total explained variance. **B.** Loadings plot of PC-1. **C.** Representative spectra of 3MH varying in concentration between 0.0058 ppm and 3 ppm (blue). **D.** PLS-R prediction plots of the test data for 3MH and used to calculate LoD using multivariate calculations. Asterisks in **B** and **C** represent the peaks chosen for the univariate calculation of the limit of detection (LoD). PLS-R models were produced using k-fold leave-one-out double cross validation. Data for figures **A – C** has been baseline corrected, smoothed and vector normalised.

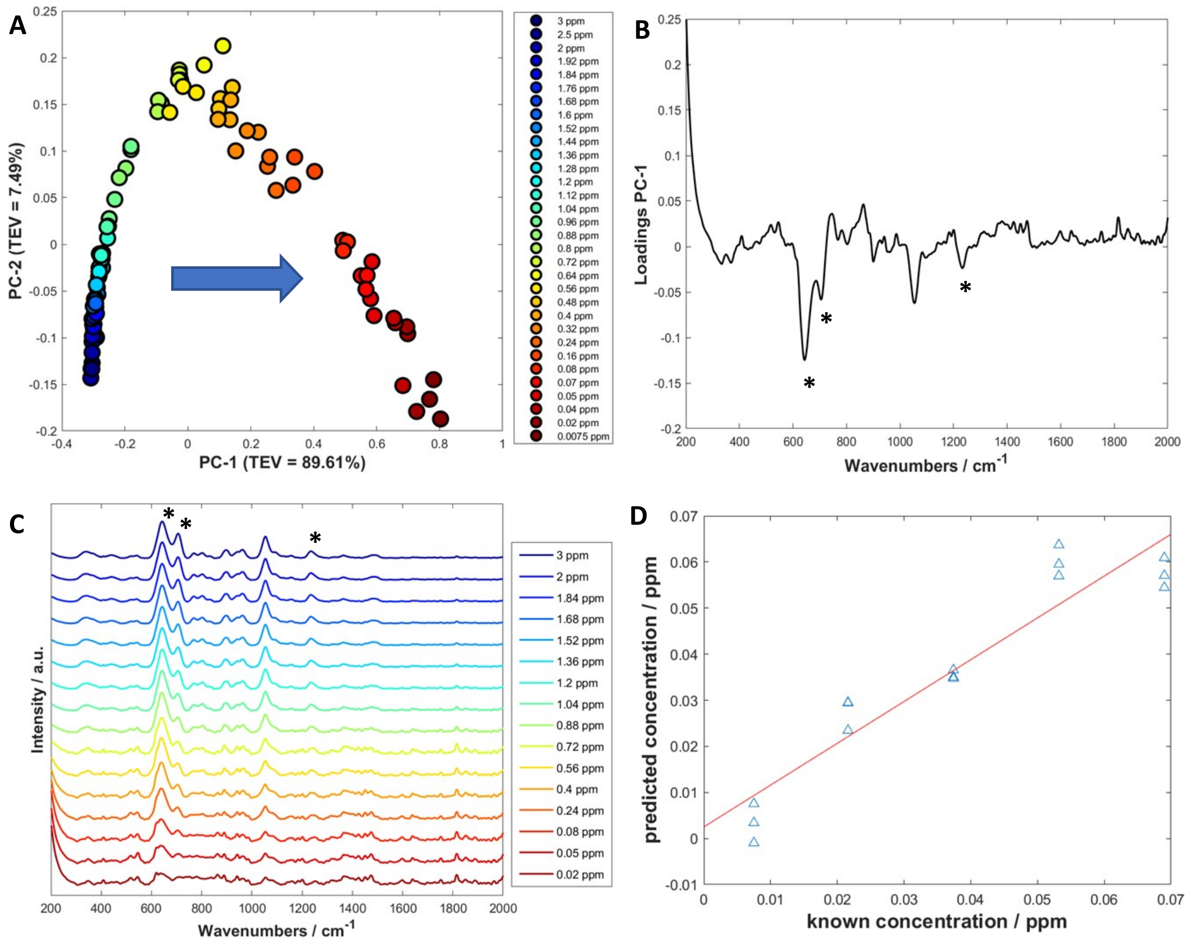

Figure S13: A. A PCA scores plot of 2M3MP at varying concentrations between 0.0075 ppm and 3 ppm. The arrow indicates direction of decreasing concentration and TEV is the total explained variance. **B.** Loadings plot of PC-1. **C.** Representative spectra of 2M3MP varying in concentration between 0.0075 ppm and 3 ppm (blue). **D.** PLS-R prediction plots of the test data for 2M3MP and used to calculate LoD using multivariate calculations. Asterisks in **B** and **C** represent the peaks chosen for the univariate calculation of the limit of detection (LoD). PLS-R models were produced using k-fold double cross validation. Data for figures **A – C** has been baseline corrected, smoothed and vector normalised.

Determining total concentration of multiple thiols in solution which equals one monolayer of analytes on the nanoparticle surface

To measure multiple thiols in solution, first, the total concentration of the thiols at which the nanoparticles are covered in one monolayer of the analytes is determined. This is because, when the total monolayer coverage exceeds one layer, there is competition at the surface depending on the binding energies of the analytes. However, if the thiol coverage concentration is equal to or below one monolayer, then both analytes have an equal opportunity to bind to the metal surface.[7] Therefore, the total concentration for one monolayer needs to be measured so that when the number of analytes in solution increases, all the analytes can be detected within the mixture.

Two thiols of equal concentration (ppm) were prepared in mixtures of total concentrations between 0.404 ppm and 100 ppm. Each thiol was tested with the three other thiols of interest, meaning six different mixtures were analysed with three replicates at each concentration. The ratio of the peak

areas were used to examine when one monolayer had been reached. This parameter was used as when the concentration of one monolayer is exceeded, the ratio of peak areas changes.

For the majority of the mixtures, one monolayer of thiols appears to be achieved at a total concentration of 10 ppm (**Figure S10A**). At lower concentrations, there is a consistent pattern of an increase in peak areas of the peaks of interest for each thiol seen in all mixtures. Between 10 ppm and 20 ppm, for most of the mixtures, there then does not appear to be much change in the peak areas. This suggests that one monolayer has been reached within this total concentration range. Above 20 ppm, the peak area ratios appeared to increase or decrease depending on the plot. This is also observed when looking at the changes in the individual peak areas of the peaks of interest. At total concentrations above 20 ppm, one peak appears to show a positive correlation of peak area with increasing total concentration and the other peak shows a negative correlation. This is due to competition at the surface due to the total concentration of thiols being greater than one monolayer.

Therefore, the concentration at one monolayer is estimated to be ~10 ppm. All other measurements of the mixtures did not exceed a total concentration of 10 ppm so that there was no competition of thiols on the surface. The concentration for one monolayer is low because the thiols are branched and therefore cannot pack as efficiently on to the nanoparticle surface in comparison to straight-chained alkane thiols. The concentrations of the individual thiols in a mixture were between 0.05 ppm and 1 ppm, so, the total concentration of the mixture never exceeded 4 ppm.

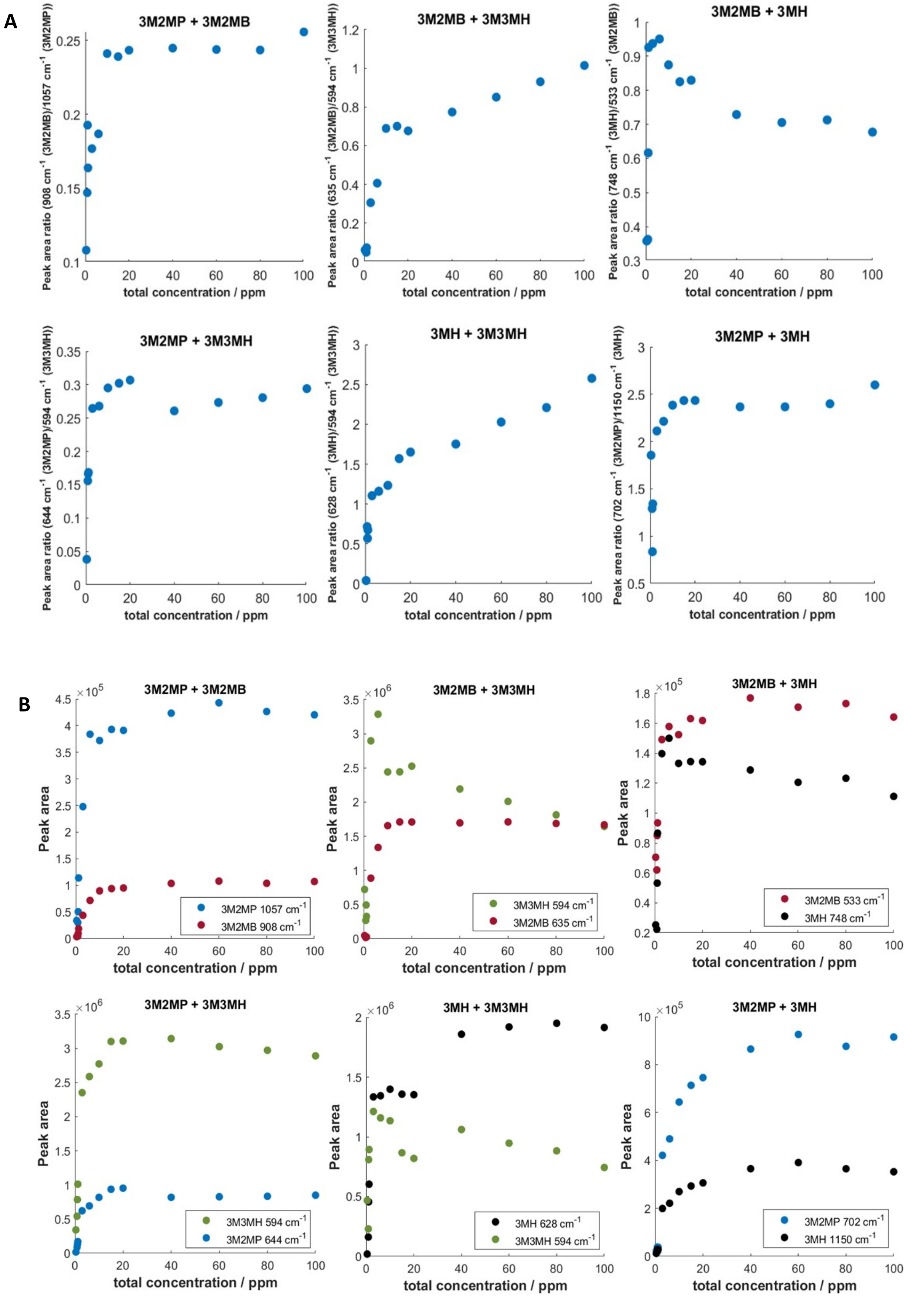

Figure S14: A. Plots of the peak area ratios of characteristic peaks for 3MH, 3M2MB, 3M3MH and 3M2MP using the six possible combinations of two of thiols. **B.** Plots of the changes of the peak areas of characteristic peaks for 3MH, 3M2MB, 3M3MH and 3M2MP used in calculating peak area ratios using the six possible combinations of two of thiols. The thiols are at the same concentration and peak areas were calculated using the raw spectra.

Examining changes in the spectrum of a mixture when varying the concentration of one thiol

To see how the spectrum of a mixture varies when thiol concentrations change, solutions containing three thiols at the same concentration and another thiol at a different concentration were prepared. This experiment was repeated at different concentrations of the fourth thiol ranging from 0.2 ppm to 2 ppm. This was done to investigate variations in the spectra at low and high concentrations of the fourth thiol in the solution.

As seen in all the spectra of the thiols, as a thiol increases in concentration with the remaining thiols at the same concentration **(Figure S11)**, different peaks start to increase in intensity causing changes in the appearance of the spectrum. For example, in the spectra where 3M3MH is changing in concentration, the peak at 596 cm^{-1} increases in intensity with increasing concentration. Furthermore, the peak at 1053 cm^{-1}, 1107 cm^{-1} and 1053 cm^{-1} increase in intensity with increasing concentration of 2M3MB, 3MH and 2M3MP respectively. There are also slight shifts in peaks in the spectrum of the mixture that are present due to overlap of peaks at similar wavelengths from different thiols. This is seen for the gauche v(C-S) peak which shifts from 695 cm^{-1} to 706 cm^{-1} with an increasing concentration of 2M3MP and the opposing shift for an increase in concentration of 3MH. Therefore, changes in the spectra can be visualised with changing concentrations of thiols in a mixture.

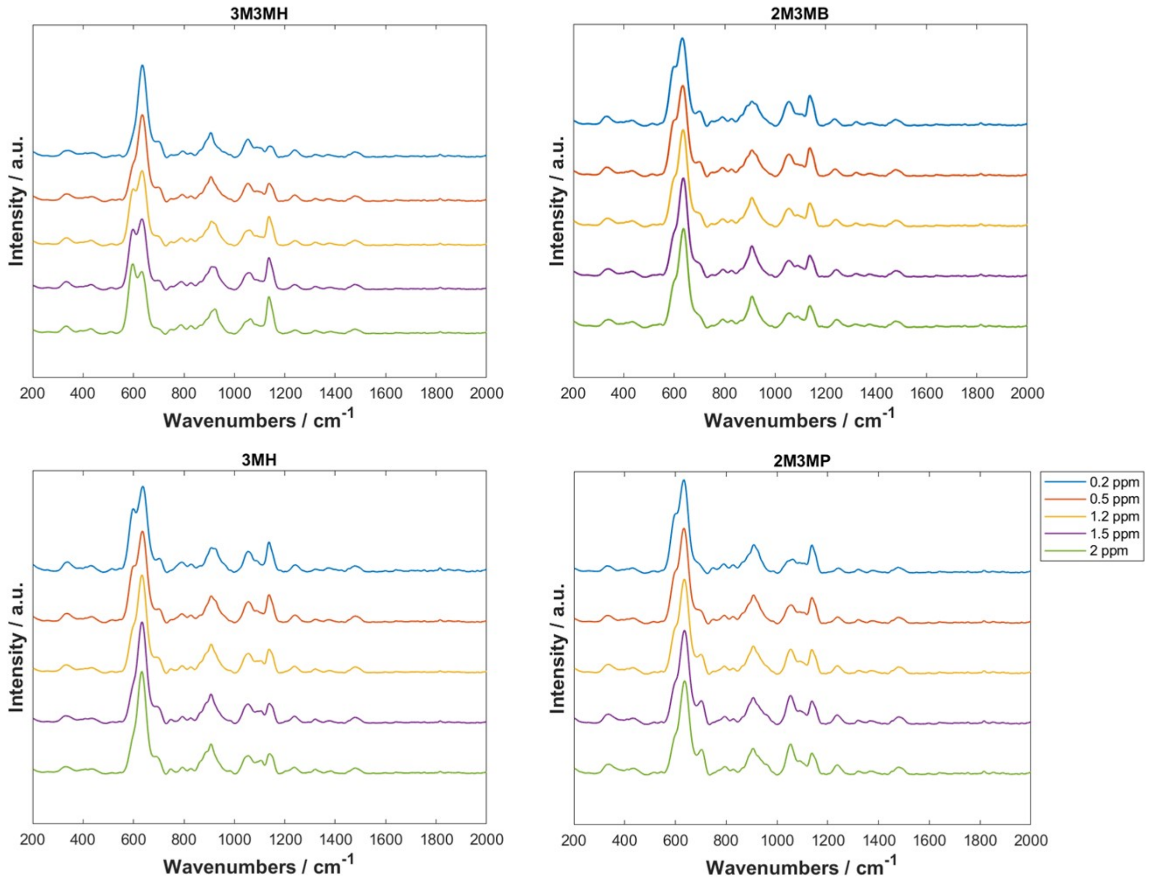

Figure S15: Spectra of three thiols remaining at a constant concentration of 0.75 ppm with the fourth thiol changing concentration across the spectra from 0.2 ppm to 2 ppm. Titles of spectra represent the thiol which is changing in concentration in the spectra. Data has been baseline corrected, smoothed and vector normalised.

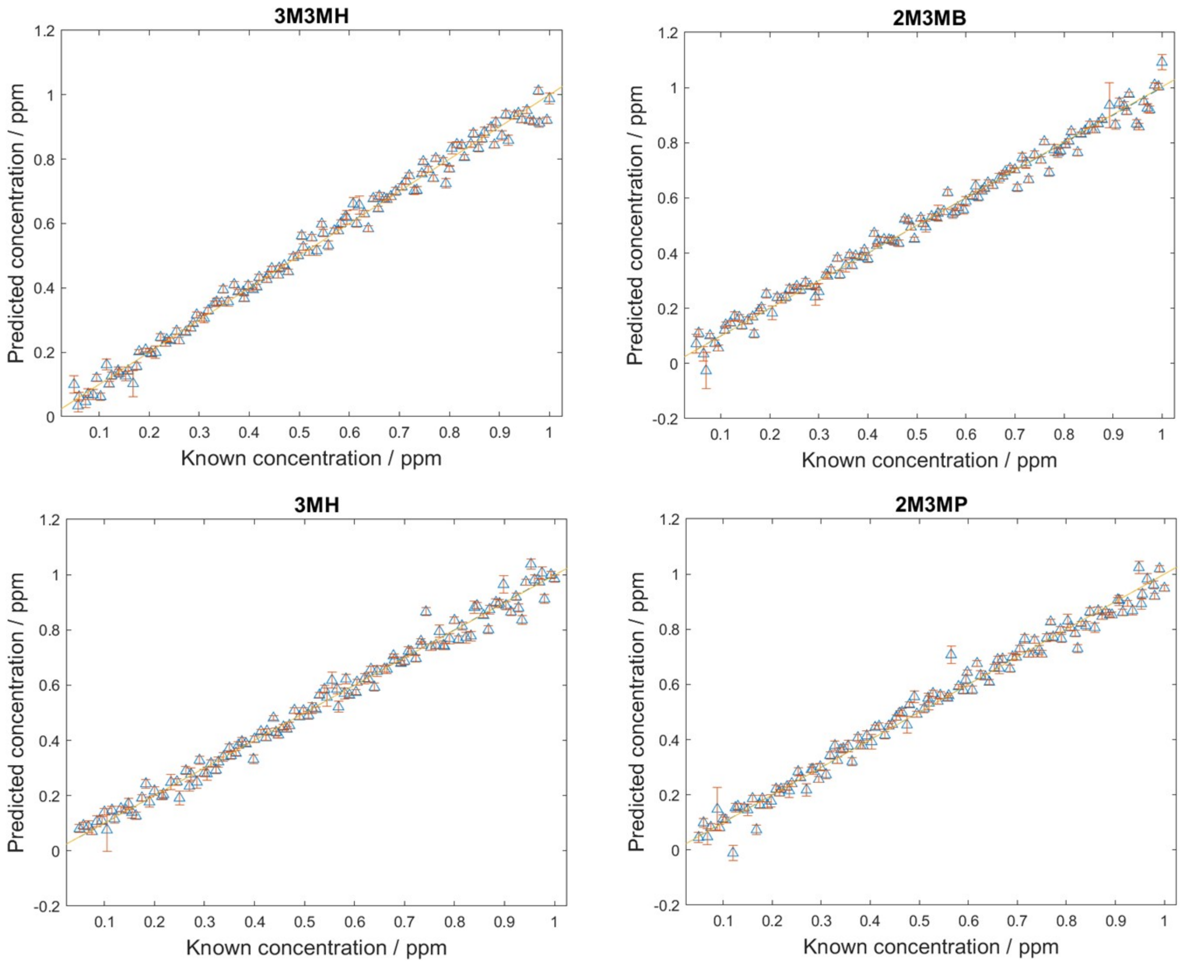

Figure S16: PLS-R PLS-1 prediction plots of the test data for each of the thiols of interest in a multiplex mixture. The PLS-R prediction were produced using 1000 bootstraps for validation, and are shown for these validation only (i.e., not for the training data).

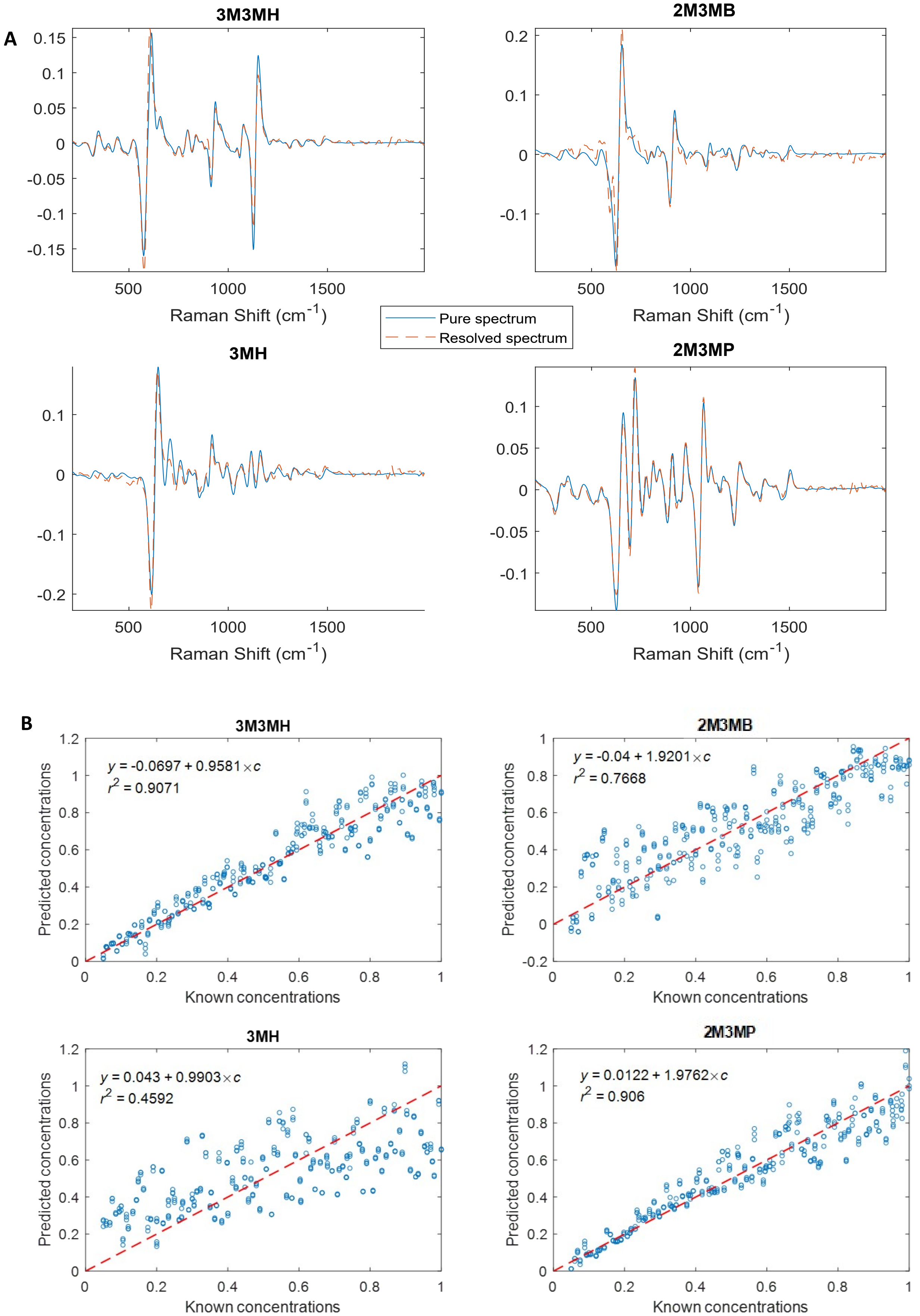

Figure S17: A. Resolved first derivative spectra of the individual thiols in a mixture using MCR-ALS. **B.** Predicted concentration plots of thiols using the resolved first derivative concentration profiles modelled using MCR-ALS.

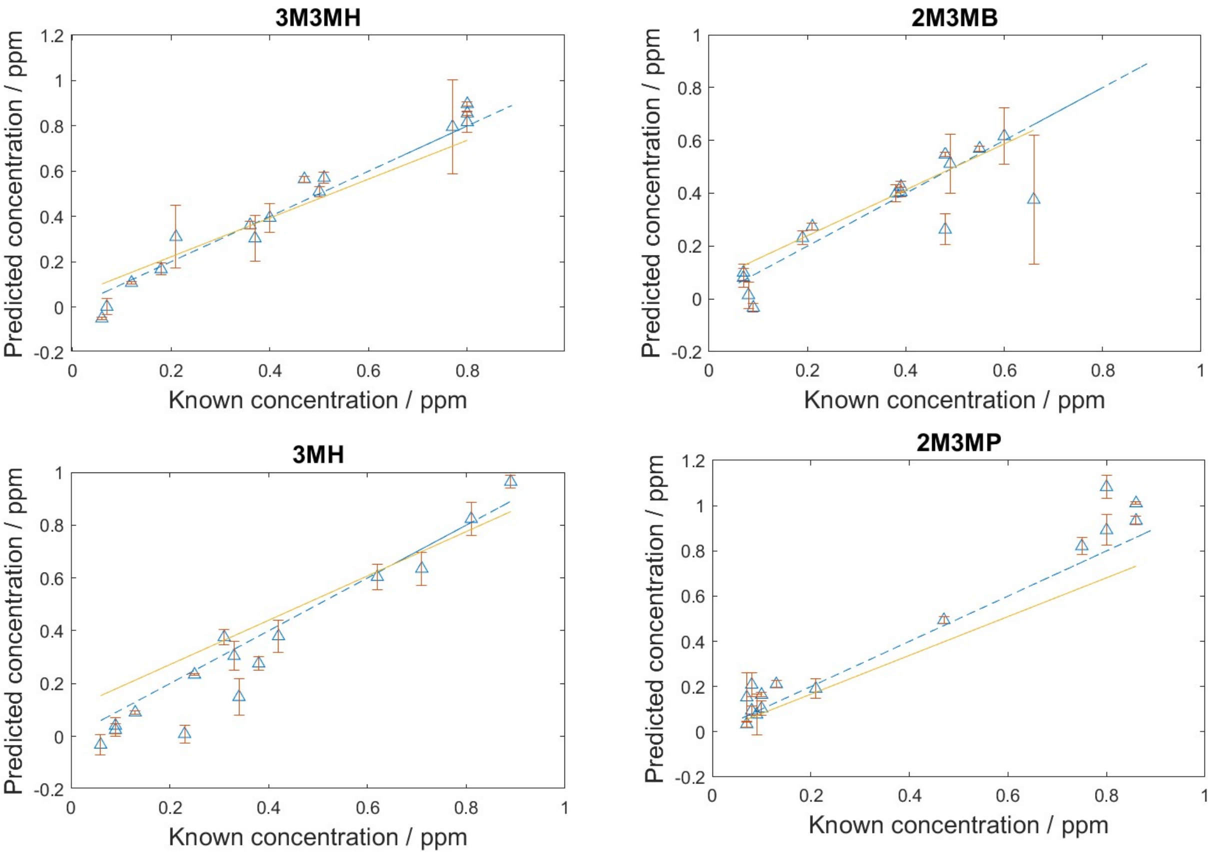

Figure S18: PLS-1 prediction plots of the blind test data for each of the thiols of interest in a multiplex mixture.

References

1. H. Fisk, C. Westley, N. J. Turner and R. Goodacre, *J. Raman Spectrosc.*, 2016, **47**, 59-66.
2. P. C. Lee and D. Meisel, *The Journal of Physical Chemistry*, 1982, **86**, 3391-3395.
3. X. Dong, H. Gu, J. Kang, X. Yuan and J. Wu, *Colloids and Surfaces A: Physicochemical and Engineering Aspects*, 2010, **368**, 142-147.
4. M. Wuithschick, A. Birnbaum, S. Witte, M. Sztucki, U. Vainio, N. Pinna, K. Rademann, F. Emmerling, R. Kraehnert and J. Polte, *ACS Nano*, 2015, **9**, 7052-7071.
5. L. Mikac, M. Ivanda, M. Gotić, T. Mihelj and L. Horvat, *J. Nanopart. Res.*, 2014, **16**, 2748.
6. R. N. Cassar, D. Graham, I. Larmour, A. W. Wark and K. Faulds, *Vibrational Spectroscopy*, 2014, **71**, 41-46.
7. A. Stewart, S. Zheng, M. R. McCourt and S. E. J. Bell, *ACS Nano*, 2012, **6**, 3718-3726.
